# Synthesis, delivery, and molecular docking of fused quinolines as inhibitor of Hepatitis A virus 3C proteinase

**DOI:** 10.1038/s41598-021-98529-0

**Published:** 2021-09-23

**Authors:** Mehrnaz Rafiei Jorshari, Manouchehr Mamaghani, Parivash Jahanshahi

**Affiliations:** Department of Chemistry, Faculty of Science, University of Guilan, Rasht, Iran

**Keywords:** Drug discovery, Drug delivery

## Abstract

It is widely accepted that Hepatitis A virus (HAV) is responsible for liver failure and even death in older people and in people with other serious health issues; so, proposing new compounds with inhibitory activity can help to treated of these disease’s. In current study, a new class of quinolines is proposed with inhibitor activity of the HAV proteinase. So, in the first step, fused quinoline derivatives has been synthesized in short reaction time (12.0 min) and high efficiency yields (94%) in presence of 1-carboxymethyl-2,3-dimethylimidazolium iodide ([cmdmim]I) ionic liquid catalyst using a new method. In the following, chemical reactivity and inhibitory activity of synthesized quinolines were evaluated in density functional theory (DFT) framework and molecular docking methodologies. High global softness (0.67 eV), low HOMO_SWBNNT_-LUMO_4a_ gap (4.78 eV), and more negative adsorption energy (− 87.9 kJ mol^−1^) in these quinolines reveal that the 4a and 4b compounds have better delivery than other quinolines using SWBNNT as suitable carrier to target cells. Molecular docking shows that the best cavity of the HAV has − 134.2 kJ mol^−1^ interaction energy involving bonding and non-bonding interactions. In fact, these interactions are between fused quinolines with especial geometries and sidechain flexibility amino acids residues inside the best binding site of the HAV, as hydrogen bonding, steric, and electrostatic interactions. So, these interactions imply that proposed fused quinolines have good inhibitor activity for the HAV.

## Introduction

Human Hepatitis A virus (HAV) is regarded as an inflammation of the liver that this infectious or epidemic hepatitis is transmitted by the fecal–oral route^1–3^. Low time between infection and symptoms, high rate of infection are strong reasons for motivations scientists whose always looking for logical solutions in order to help to liver disease^4,5^. Albeit, it should not be forgotten that drug design and production are time consuming and complicated. In order to decrease these issues, chemistry research groups partially help with suggestion of design and synthesis new compounds with anti-virus properties. In current study, we propose derivatives of a category of organic compound called fused quinoline.

Quinolines are an important group of heterocyclic compounds that have significant biological properties^6–10^. In fact, these compounds are used as potential anti-cancer, anti-tumor, and anti-malarials agents^11–13^. There are several proposed methods to synthesis of these compounds^14–37^, such as Povarov reaction^14^, radiation to second type amines^15^ and so on^14–40^. For example, for the first time, interaction of quinolines and solid support was carried out using microwaves with solvent free technique^6^. In another study, compounds based on quinoline were proposed as inhibitor production of the envelope glycoprotein in Dengue Virus Serotype 2 and DNA gyrase through direct binding to the bacterial chromosome^40^. Also, these compounds were designed and synthesized for DNA-gyrase and topoisomerase-II inhibition^41^. Another series of quinolines has been prepared as MEK (MAP kinase kinase) inhibitors^42^. In addition, synthesized quinolines via the Mannich reaction were prepared against Gram-positive (G+), Gram-negative (G−) bacteria^39^.

Although above mentioned studies have proposed methods with the aim using into pharmaceutical industries, they have low relative yield and high reaction time. So, in current study, we propose a novel method to synthesis of fused quinolines with excellent yield and short reaction time using 1-carboxymethyl-2,3-dimethylimidazolium iodide ([cmdmim]I) ionic liquid catalyst^43^. In fact, the [cmdmim]I as one of recyclable catalysts mainly results in straightforward work-up procedure, and reusability of the catalyst in about 5 consecutive runs without any appreciable decrease in activity^43^. In fact, ionic liquids have important role into synthesis of these compounds on pharmaceutical industries. On the other hands, we are going to study quantum mechanics calculations due to introduce suitable quinolines with high chemical reactivity. In addition, their inhibitory activity in interaction with the HAV will study using molecular docking computations.

## Experimental section

### General

Chemicals were purchased from the Merck chemical companies. Extend of reactions were evaluated by thin layer chromatography (TLC) covered using silica-gel 60, F256 contracted by Merck chemical companies. In the TLC chromatography was used from *n*-Hexane and Ethyl Acetate solvents. Melting point was recorded using electro thermal devices. FT-IR, ^1^H-NMR and ^13^C-NMR spectra were recorded using VERTIX 70 Brucker, a Brucker (400 MHz) Advance DRX in DMSO-d6 solvent. Chemical shift (in ppm) were investigated in related to tetra methyl silane (TMS) as internal standard.

### Preparation of the [cmdmim]I ionic liquid

The [cmdmim]I ionic liquid is high impact catalyst with solid state and orange color; whose previously synthesized by our research group^43^.

### Synthesis of the 7-(3-Nitro-Phenyle)-H8-Benzo [h] Indo [1.2-b] quinoline-8-on (4a)

In balloon flask (100 mL) equipped with an electrical condenser, a mixture consisting of 1, 3-indandeion (0.146 g, 1 mmol), 3-Nitro Benzaldehide (0.1510 g, 1 mmol), and 1-Naphtyle Amine (0.143 g, 1 mmol), in presence of the [cmdmim]I ionic liquid catalyst (10% molar), water solvent (10.0 mL) was heated for 15 min at reflux conditions. The progress of the reaction was followed by the TLC (eluting with a mixture of Petroleum Ether, Ethyl Acetate (3:10), accompanied one drop of Methanol). After cooling and solvent evaporation, the mixture of reaction was dissolved in Ethanol, Ethyl Acetate, and Chloroform (30:30:10 mL) and then, it was purified and dried. The yellow powder of the synthesized compound (4a; 0.361 g) was produced in 90.0% yield and 326–328 °C. Other quinoline derivatives are produced using proposed method, see Table S1 and S2 (S means supplementary file).

The 4a synthesis has been carried out in various conditions such as six different solvents, 10% molar of catalyst, and reflux condition, see Table S3. Influence of different catalyst and their value have been evaluated after investigation of reliable solvent, see Tables S2 to S5. According to Tables S4 and S5, the most favorable condition for the 4a synthesis was using 10% molar catalyst in water solvent and reflux condition.

To confirmation of produced structures, 4a, the IR, ^1^H-NMR and ^13^C-NMR spectroscopies were used. IR (KBr): 3062 (C–H stretch, aromatic), 1710 (C=O stretch), 1639 (C=N stretch), 1608, 1571, 1469 (C–C stretch, aromatic), 1519, 1346 (NO2 stretch), 844, 815, 763, 730 (C–H out of plane bending, aromatic) cm^−1^. ^1^H-NMR (400 MHz, DMSO) δ (ppm): He or Hh 9.46 (dd, J = 2 and 8.2 Hz, 1H), He or Hh and Hk 8.47–8.50 (m, 2H), Hl 8.27 (d, J = 7.2 Hz, 1H), Ha or Hd 8.04 (d, J = 7.6 Hz, 1H), Hj 7.98 (d, J = 8.8 Hz, 1H), Hc 7.93 (t, J = 6.2 Hz, 1H), Hm, Hb and Hf or Hg 7.86–7.93 (m, 3H), Hn 7.73 (d, J = 7.2 Hz, 1H), Hg or Hf 7.66 (t, J = 7.2 Hz, 1H), Hi 7.52 (d, J = 8.8 Hz, 1H). ^13^C-NMR (100 MHz, DMSO-d6) δ (ppm): C=O ketone 192, 24 aromatic Carbone: 162.5, 160.5, 154.2, 150.7, 148.4, 146.5, 144.0, 141.9, 139.9, 139.7, 137.6, 135.9, 135.4, 133.1, 131.3, 129.1, 128.7, 128.3, 125.6, 125.0, 124.6, 124.5, 123.6, 122.9, and 121.9.

### Theoretical section

Low lying structure have been investigated in the framework of hybrid-DFT (H-DFT) by using Gaussian09 program package^44^. The ωB97-XD/6-31g(d) level of theory was selected using DFT calibration method^45,46^; which combines long range correlation functionals, with all electron basis set, 6-31g(d)^47^. Geometry optimization of structures carried out without any symmetry constrains (C1 symmetry). Vibrational frequency test guarantees that the optimized geometries are in real minima.

Tendency and role of the SWBNNT, the 4a, and derivatives in their interactions have been investigated using global chemical reactivity descriptors such as chemical potential (μ = − (IE + EA)/2), hardness (η = (IE − EA)/2), softness (S = 1/η), and electrophilicity (ω = μ2/2η)^48^. In fact, the μ is the escaping tendency of electrons, the η is a resistance to charge transfer, the ω is floating of electron between nucleophile and electrophile. The IE and EA are adiabatic ionization energy and electron affinity, respectively; they calculated using tree approach point methods:

1 $$ {\text{IE}}_{ad} = {\text{E}}_{total} \;(compound^{ + } ) - {\text{E}}_{total} \;(compound) $$

2 $$ EA = {\text{E}}_{total} \;(compound) - {\text{E}}_{total} \;(compound^{ - } ) $$

where ${\text{E}}_{total} \;(compound)$, ${\text{E}}_{total} \;(compound^{ + } )$, and ${\text{E}}_{total} \;(compound^{ - } )$ are total energy of neutral, cationic, and anionic compounds (SWBNNT and fused quinoline derivatives), respectively. Thermodynamic stability of complexes was calculated using adsorption energy equation:

3 $$ {\text{E}}_{ads} = {\text{E}}_{{SWBNNT/4{\text{a}}}} - ({\text{E}}_{SWBNNT} + {\text{E}}_{{{\text{fused }}\;{\text{quinoline }}\;{\text{derivatives}}}} ) $$

where ${\text{E}}_{{SWBNNT/4{\text{a}}}}$, ${\text{E}}_{SWBNNT}$, and ${\text{E}}_{{{\text{fused }}\;{\text{quinoline }}\;{\text{derivatives}}}}$ are total energy of complexes, SWBNNT, and ${\text{fused}}\;{\text{ quinoline }}\;{\text{derivatives}}$ molecule, respectively. In order to achievement to realistic value of the E_ads_, zero-point vibrational energy (ZPVE) and basis set supper position error (BSSE) was considered. Therefore, the counterpoise correlation was used to correct BSSE error. Geometric information is completed by results of natural bond orbital (NBO) analysis. Type of bonds, depletion of occupancies, percent of Lewis and non-Lewis and stabilization energy (${\text{E}}_{{{\text{ij}}}}^{2}$) are obtained by NBO analysis:

4 $$ {\text{E}}_{{{\text{ij}}}}^{2} = \frac{{\left| {\left\langle {{\text{i}}|{\hat{\text{H}}}|{\text{j}}} \right\rangle } \right|^{2} }}{{{\text{E}}_{{\text{j}}} - {\text{E}}_{{\text{i}}} }} $$

where ${\hat{\text{H}}}$ is interaction Hamiltonian, ${\text{E}}_{{\text{j}}}$ and ${\text{E}}_{{\text{i}}}$ are orbital energies, and $\left\langle {{\text{i}}\left| {\widehat{{\text{H}}}} \right|{\text{j}}} \right\rangle$ is matrix element.

### Molecular docking

**Figure 1:**
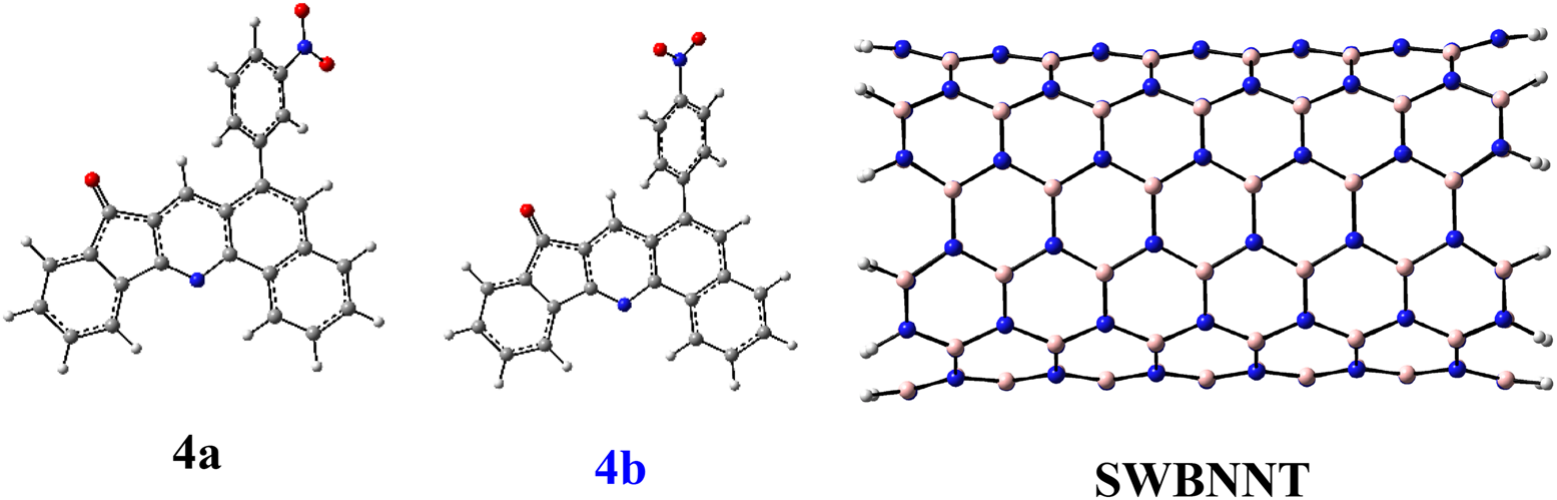
Ground state structure of the 4a, 4b, and single walled boron nitride nanotube (SWBNNT) in ωB97-XD/6-31g* level of theory.

Secondary structures of Hepatitis A virus (PDB ID: 1HAV) were retrieved from the Research Collaboratory for Structural Bioinformatics (RCSB) Protein Data Bank (http://www.rcsb.org/), see Figure S4. In geometry minimization processing, the best of their potential ligand binding site (cavity) has X: 11.96, Y: 5.87, Z: 10.08 Cartesian coordinate, radius 15 Å, volume 1438.2 Å^3^, and surface 2420.5 Å^2^, respectively. Maximum global minimization step for molecular docking was set at 1500 steps. The side chain flexibility of the amino acid residues of these proteins inside the cavity with tolerance of 1.00 and strength of 0.90 were considered, see Fig. 1.

Then, molecular docking was simulated using Molegro Virtual Docker (MVD) 6.01^49^. For this purpose, MolDock algorithm as docking method based on guided differential evolution (combination differential evolution optimization technique with cavity prediction algorithm) and force field based scoring function [extension of Piecewise Lenear Potential (PLP)]^50^ identify the potential binding site of proteins and binding orientation of ligands^50^. Re-ranking of top ranked conformations mainly results in improvement of docking accuracy^51^. The MolDock scoring function iteration was set 1500 with a simplex evolution size of 50 and a minimum of 10 runs. In addition, the simplex evolution was set for 300 steps with a neighbor distance factor of 1.00. The MolDock grid score was set with a grid resolution of 0.30 Å.

## Results and discussion

**Scheme 1:**
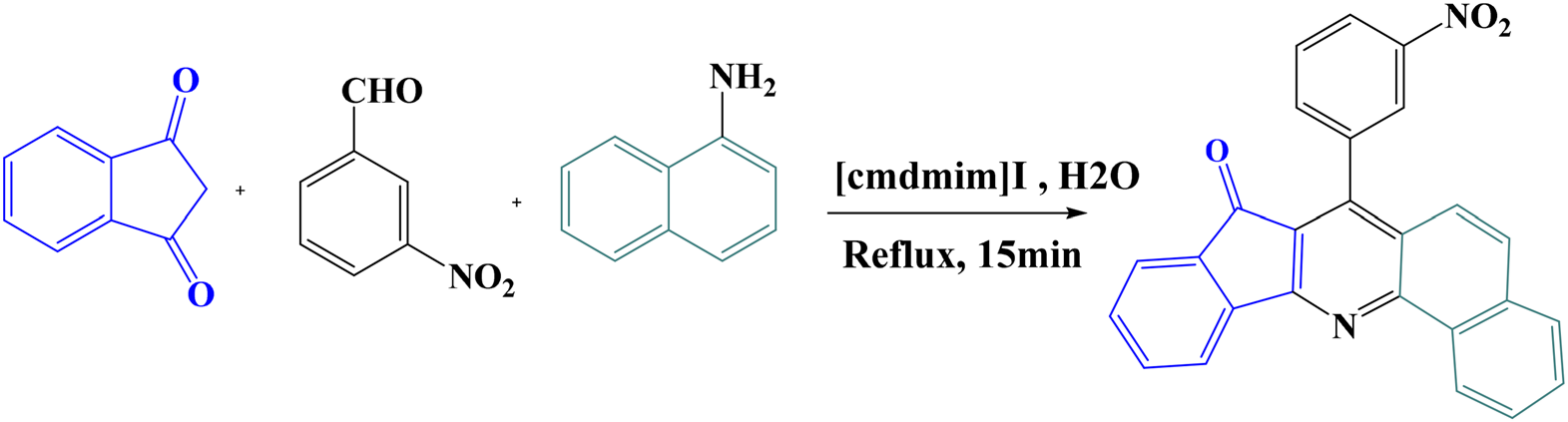
The synthetic pathway toward synthesis of 4a compound in reflux condition.

In starting point, optimization processing of reaction condition for synthesis of Indo-quinoline derivatives was carried out in different solvent and catalysts; see Table S1. The most efficiency yields (90.0%) was obtained in 10% molar the [cmdmim]I ionic liquid catalyst and reflux condition in the water as green solvent. This efficiency mainly results from selection of the 1,3-indandione, aryl aldehydes and 1-naphthylamine with the same ratio in one pot reaction, see Scheme 1.

In the proposed reaction mechanism, Scheme S1, aryl aldehydes are the key component in this reaction due to especial geometry. More importantly, role of ionic liquid in proposed mechanism (Scheme S1) is activation of aldehyde and ketone carbonyl and facilitating of reaction cyclo addition step, respectively, see Scheme S2.

The main aim of current study is to provide a conceptual theoretical framework based on ωB97-XD/6-31g* method. Geometry of quinoline derivatives in the real minima are given in Fig. 1 and Figure S1. In fact, synthesized fused quinolines are susceptible non-covalent interactions.

**Figure 2:**
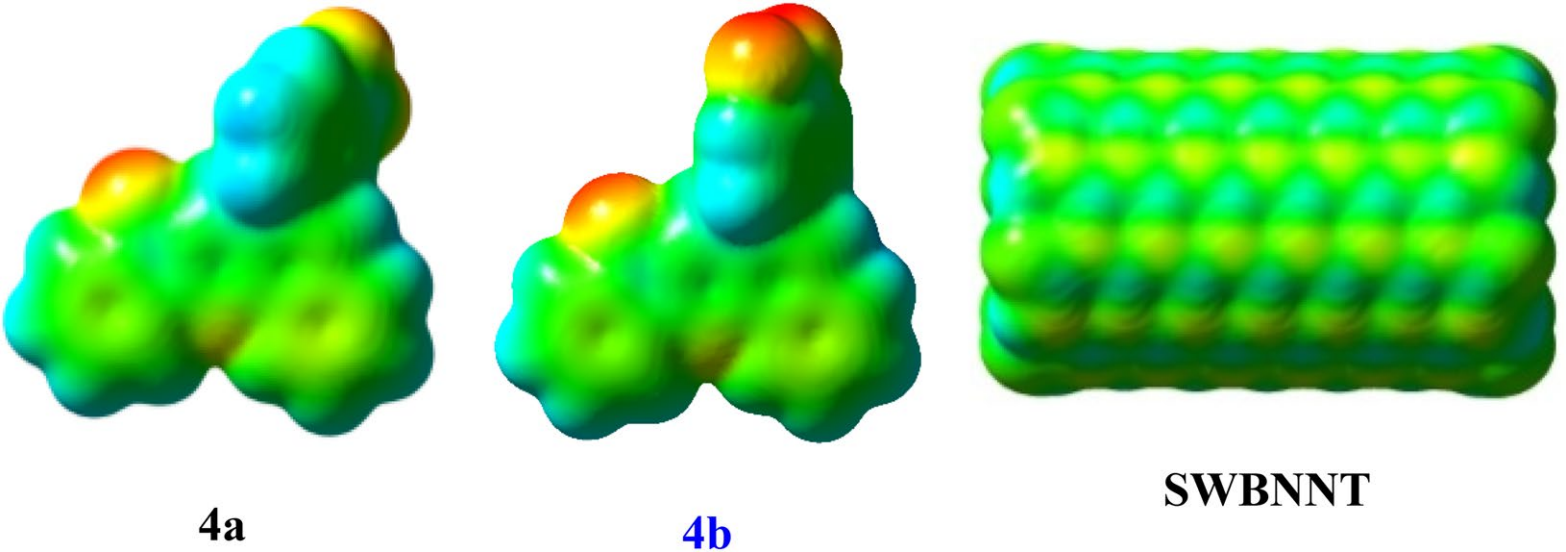
Molecular electrostatic potential (MEP) maps of the 4a, 4b, and single walled boron nitride nanotube (SWBNNT) in ωB97-XD/6-31g* level of theory.

High electron density in the molecular electrostatic potential (MEP) maps^52^, confirms non bond interaction, as shown in Fig. 2 and Figure S1. This property can give into inhibitory activity on Hepatitis A virus. The MEP maps^52^, investigate and predict active sites and strength of interactions in the 4a, derivatives and SWBNNT. According to this figure, despite areas without electron density (green color) in the 4a and SWBNNT, Oxygen and Nitrogen centers in the 4a molecule show negative electrostatic potential. In fact, these parts with electron delocalization can introduce as involve sites in their interactions.

The more charge transfer is carried out from SWBNNT to 4a molecule due to lower the HOMO_SWBNNT_-LUMO_4a_ gap than others, see Figure S2. This issue confirms by 125 kcal mol^−1^ in total second order stabilization energy.

With investigating of their role, they were interacted in suitable orientations, see Figure S3. Energy of stable configurations was considered to calculate of adsorption energy (E_*ads*_). Based on the more negative value of the E_*ads*_, 4a and 4b complex has adsorption strength and stable configuration than others, as shown in Table S8.

Density of state (DOS) analysis has been carried out due to better understanding of these interactions. Total DOS of the 4a molecule, SWBNNT, and outside complexes are given in Figure S1. The reduction of energy gaps in complexes can be attributed to better interactions. These interactions may influence on electron excitation and excited states spectrum.

**Figure 3:**
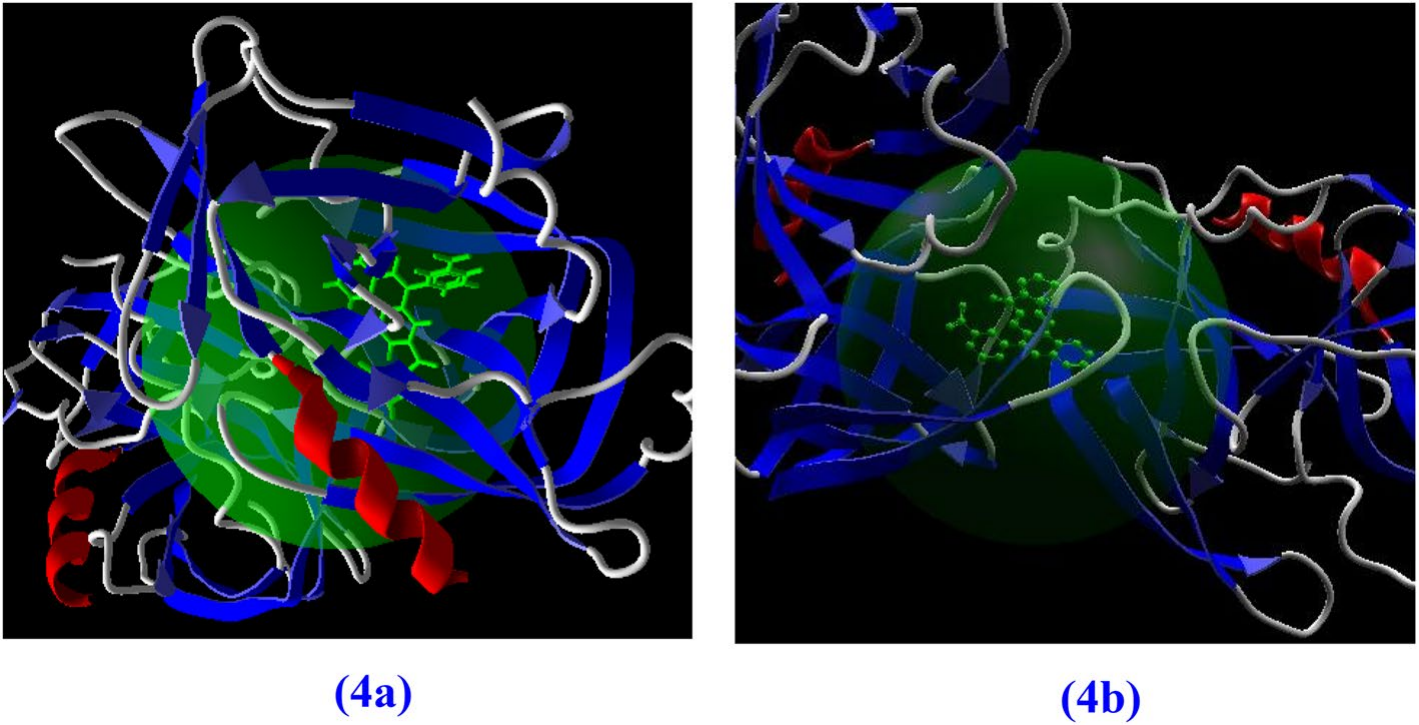
Molecular simulation of the 4a, 4b, and Hepatitis virus.

After delivery of drug molecule to target cells involving Hepatitis A virus, their interactions are important. The best geometry of docking has more negative value in Gibbs free energy at 25 ns, see Fig. 3. The best favorable sites of fused quinoline rings as drug molecule interact with Hepatitis A virus from the hydrogen bonding, electrostatic, and steric interactions.

### Molecular docking

**Figure 4:**
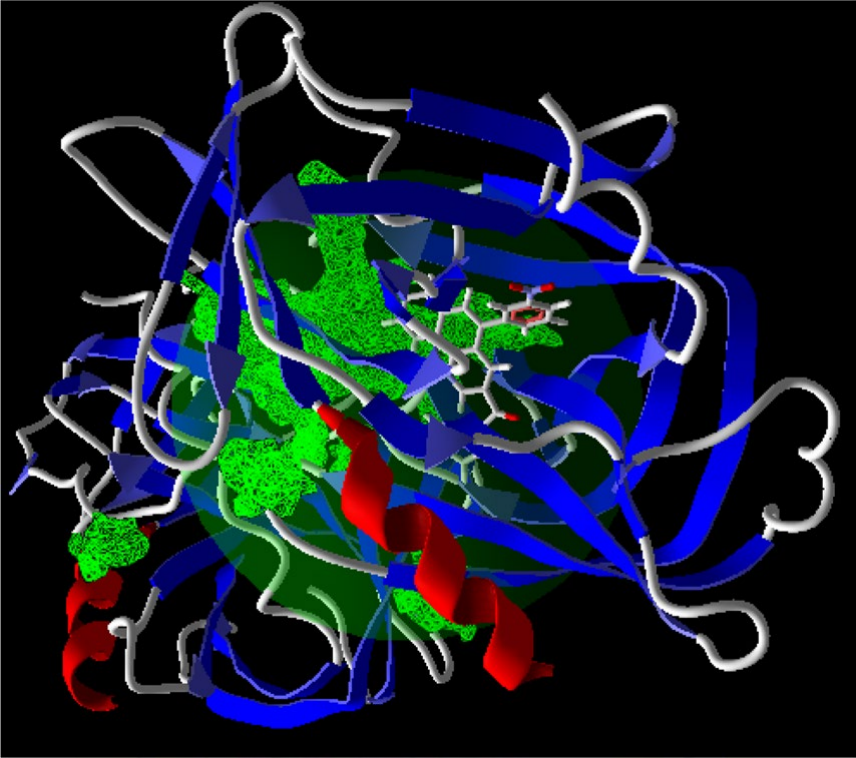
The potential ligand binding cavities of Hepatitis virus.

**Figure 5:**
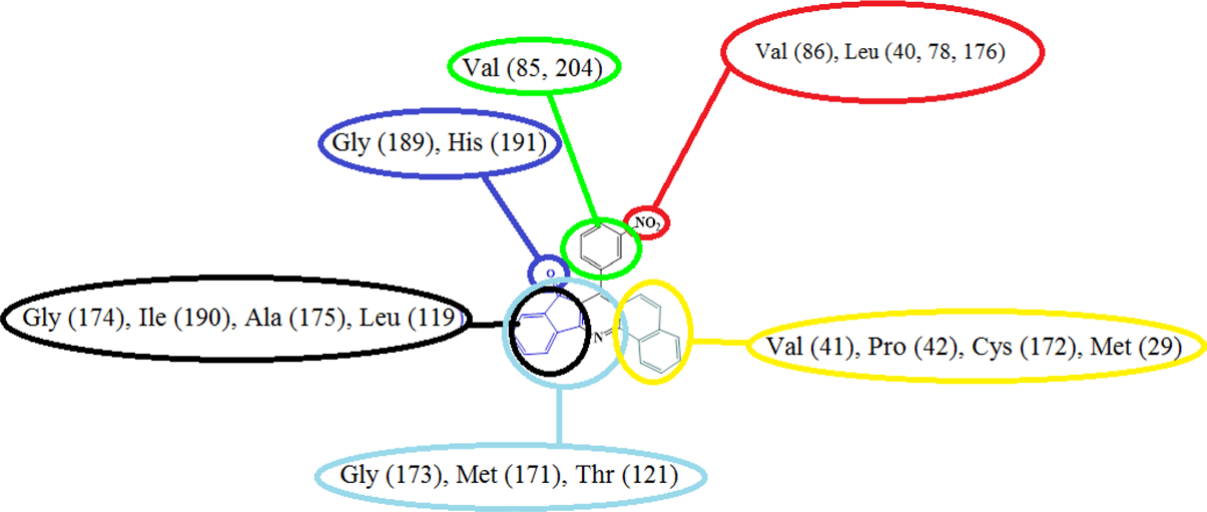
Molecular interaction between fused quinoline (4a, 4b) and active site of Hepatitis virus.

**Figure 6:**
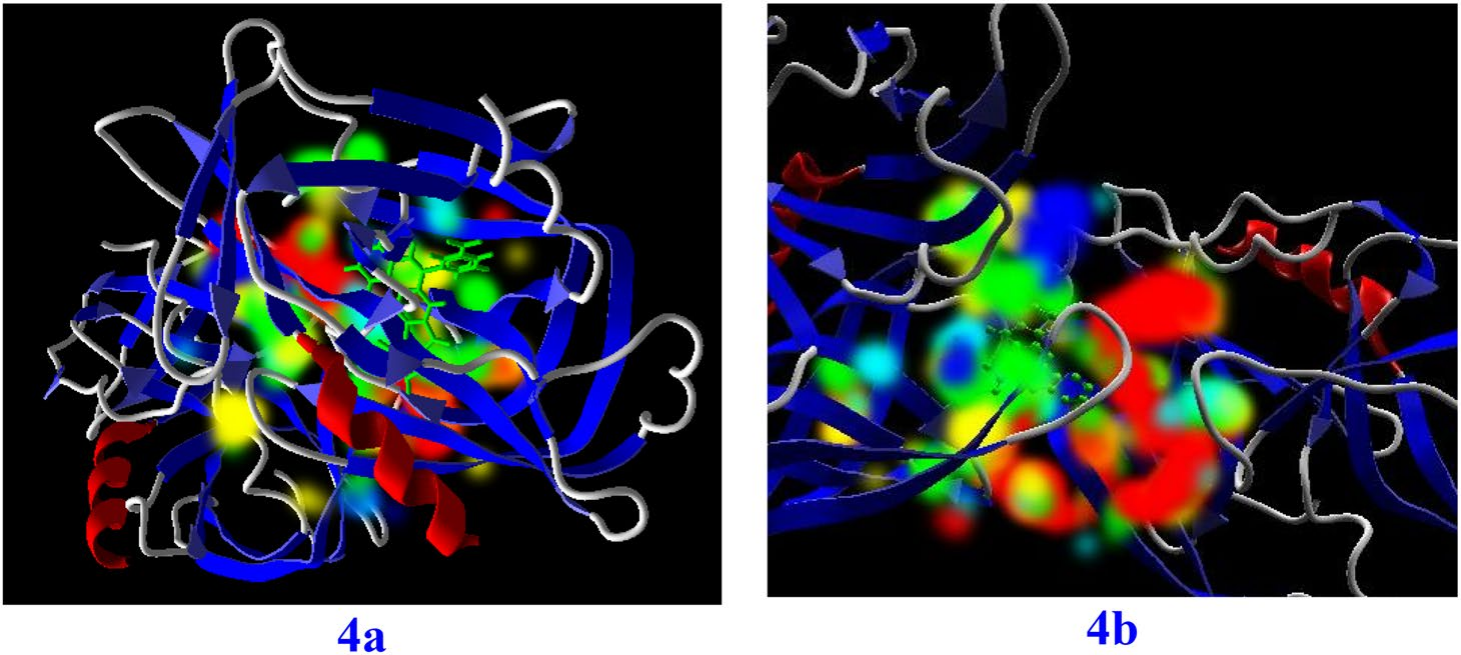
Energy map of fused quinoline (4a, 4b) at the binding cavity of Hepatitis virus.

The best binding cavity for each molecular docking simulation using the MVD program package are shown in Fig. 4. Docking scoring results in best selected cavity are presented in Table 1. More negative re-rank score showing better docking of ligand into active site of Hepatitis A virus. According to this table, re-rank score in Hepatitis A virus is negative. Molecular interaction energy between ligand and Hepatitis A virus is − 134.6 kJ mol^−1^. Besides, deep bonded and non-bonded interaction ligand inside the binding pocket of Hepatitis A virus indicating strong molecular interaction involving both interactions. Although these interactions are with Val (41, 85, 86, 204), Leu (40, 78, 119, 176), Gly (173, 174, 189), Met (29, 30), His (191), Ile (190), Ala (175), Thr(121), Cys (172), Pro (42) residues of Hepatitis A virus. These interactions at the active site of ligand are presented in Fig. 5. Indeed, introduced active sites of proteins have high-affinity for binding into ligand molecule at binding region. This result is in good agreement with chemical reactivity descriptors. Green, turquoise, yellow, red and blue colors in the energy map of Hepatitis virus might contribute in favorable steric interaction, hydrogen acceptor, hydrogen donor, electrostatic potential with the ligand^51^. While steric, hydrogen donor and electrostatic potential interaction are the most probably interaction in Hepatitis A virus, steric interaction is the most probably interaction, see Fig. 6. So, these results imply favorable ligand–protein interaction energy at the binding cavity of Hepatitis virus. In addition, it can be proposed this ligand with relatively good inhibitor.

**Table 1:** Molecular docking score of Hepatitis A virus with ligand (4a and 4b) in the favorable cavity.

Protein–ligand	RS	E_tot_	IE	E_HB_
HAV-4a	− 43.31	− 134.60	− 16.76	− 5.23
HAV-4b	− 39.02	− 127.04	− 13.43	− 3.23

*RS* Re-rank score is linear combination of internal (Steric, Van der Waals, Hydrogen bonding, and Electrostatic) and external (Torsion strain, Torsion strain sp^2^-sp^2^, Hydrogen bonding, Van der Waals, and Electrostatic) energies. *E*_*tot*_ total interaction energy between protein and pose, *IE* internal energy of pose, *E*_*HB*_ hydrogen bonding energy.

## Conclusion

In summary, one pot three component reaction in presence mentioned ionic liquid catalyst and condition is good strategies to synthesis of fused quinolines with excellent efficiency. As well as that, using of this catalyst satisfies green chemistry aims. SWBNNT is introduced as a good carrier to transform synthesized compounds without side effects. With entrance of drug molecule into target cell involving Hepatitis virus, fused quinolones interact from oxygen and nitrogen sites. So, introduced fused quinolines are susceptible these interactions due to existence five aromatic rings.

## Supplementary Information

Supplementary Information.
