# Supplementary material for: Synthesis, delivery, and molecular docking of fused quinolines as inhibitor of Hepatitis A virus 3C proteinase
Source: Sci Rep. 2021 Sep 23;11:18970. doi: 10.1038/s41598-021-98529-0 (PMC8460796; doi:10.1038/s41598-021-98529-0)
Supplement: Supplementary file 1 — Supplementary Information. [file 41598_2021_98529_MOESM1_ESM.docx]

**Synthesis, delivery, and molecular docking of fused quinolines as inhibitor of Hepatitis A virus 3C proteinase**

**Mehrnaz Rafiei Jorshari^1a^, Manouchehr Mamaghani^*, 1b^, Parivash Jahanshahi^1c^**

*^a^Department of Chemistry, Faculty of Science, University of Guilan, Rasht, Iran.*

**Corresponding author: Email: m.mamaghani2020@yahoo.com*

**Table S1.** Synthesis of Indeno quinoline derivatives using (cmdmim)I ionic liquid catalyst.

| **Product** | **Color of powder** | **Melting point (°C)** | **Time (min)** | **Randeman (%)** |
| --- | --- | --- | --- | --- |
| 4a | Yellow | 326.0-328.0 | 15.0 | 90.0 |
| 4b | Yellow close to green | 330.0-332.0 | 15.0 | 85.0 |
| 4c | Yellow | 323.0-325.0 | 18.0 | 85.0 |
| 4d | Yellow | 247.0-249.0 | 17.0 | 95.0 |
| 4e | Yellow close to green | 268.0270.0 | 25.0 | 90.0 |
| 4f | Yellow | 280.0-282.0  (279.0-281.0)* | 17.0 | 85.0 |
| 4g | Yellow | 266.0-268.0 | 20.0 | 81.0 |
| 4h | Yellow | 260.0-262.0 | 18.0 | 86.0 |
| 4i | Yellow | 239.0-241.0 | 18.0 | 78.0 |
| 4j | Yellow | 255.0-257.0  (259.0-261.0)* | 15.0 | 86.0 |
| 4k | Yellow | 275.0-277.0 | 18.0 | 82.0 |
| 4l | Yellow | 285.0-287.0  (289.0-291.0)* | 18.0 | 82.0 |

*Ref (^1^).

**Table S2.** Comparison of proposed method for synthesis of 7-(4-choloro Phenyl) Aryl-H8-Benzo [h] Indeno [1, 2 b] quinoline-8-on in presence of (cmdmim)I ionic liquid catalyst with other used method.

| **catalyst** | **Value (%mol)** | **Solvent** | **Temperature (°C)** | **Time (min)** | **Randeman (%)** | **Ref** |
| --- | --- | --- | --- | --- | --- | --- |
| (cmdmim)I | 10.0 | Water | Reflux | 15.0 | 86.0 | Proposed |
| 3-Bromo Melamine | 15 | Free solvent | 80 | 45 | 95 | (^2^) |
| -* | - | Free solvent | 60.0 | 17.0 | 83.0 | (^1^) |

*In Ultra sound condition.

**Table S3.** Effect of different solvent in yield and time of 7-(3-Nitro-Phenyle)-H8-Benzo [h] Indeno [1.2-b] quinoline-8-on synthesis.

| **Solvent** | **Time (min)** | Yield **(%)** |
| --- | --- | --- |
| Ethanol | 26.0 | 71.0 |
| Methanol | 30.0 | 52.0 |
| Water | 15.0 | 90.0 |
| Ethylene-Glycol | 20.0 | 82.0 |
| Aceto Nitryl | 22.0 | 47.0 |
| Free solvent | 30.0 | Very, very low |

**Table S4.** Effect of different catalyst in yield and time of 7-(3-Nitro-Phenyle)-H8-Benzo [h] Indeno [1.2-b] quinoline-8-on synthesis in reflux condition, (solvent: Water, 10% molar catalyst).

| **catalyst** | **Time (min)** | Yield **(%)** |
| --- | --- | --- |
| p-Toluene Sulfonic acid | 18.0 | 68.0 |
| $\mathrm{ZrO}_{2}$ | 35.0 | 52.0 |
| [cmdmim]I | 15.0 | 90.0 |
| Acetic acid-Glycial | 26.0 | 73.0 |
| Imidazole | 40.0 | 70.0 |

**Table S5.** Effect of catalyst value in yield and time of 7-(3-Nitro-Phenyle)-H8-Benzo [h] Indeno [1.2-b] quinoline-8-on synthesis in water solvent and reflux condition.

| **catalyst value (%molar)** | **Time (min)** | Yield **(%)** |
| --- | --- | --- |
| 5 | 22.0 | 72.0 |
| 10 | 15.0 | 90.0 |
| 15 | 15.0 | 90.0 |

**Proposed reaction mechanism**

Role of ionic liquid in proposed mechanism is activation of aldehyde and ketone carbonyl and facilitate the formation of the cyclic, respectively, see scheme S1.

**Scheme S1.** Mechanism of 7-Aryl-H8-Benzo [h] Indeno [1, 2 b] quinoline-8-on in presence of (cmdmim)I ionic liquid.

To confirmation of produced structures, **4a**, the IR, ^1^H-NMR, and ^13^C-NMR stereoscopies were used. IR (KBr): 3062 (C-H stretch, aromatic), 1710 (C=O stretch), 1639 (C=N stretch), 1608, 1571, 1469 (C-C stretch, aromatic), 1519, 1346 (NO_2_ stretch), 844, 815, 763, 730 (aromatic C-H out of plane bending) cm^-1^. ^1^H-NMR (400 MHz, DMSO) δ (ppm): $H_{e}$ or $H_{h}$ 9.46 (dd, *J*= 2 and 8.2 Hz, 1H), $H_{e}$ or $H_{h}$ and $H_{k}$ 8.47-8.50 (m, 2H), $H_{l}$ 8.27 (d, *J*= 7.2 Hz, 1H),$H_{a}$ or $H_{d}$ 8.11 (dd, *J*= 2 and 7.8 Hz, 1H), $H_{a}$ or $H_{d}$ 8.04 (d, *J*= 7.6 Hz, 1H), $H_{j}$ 7.98 (d, *J*= 8.8 Hz, 1H), $H_{c}$ 7.93 (t, *J*= 6.2 Hz, 1H), $H_{m}$, $H_{b}$ and $H_{f}$ or $H_{g}$ 7.86-7.92 (m, 3H), $H_{n}$ 7.73 (d, *J*= 7.2 Hz, 1H), $H_{g}$ or $H_{f}$ 7.66 (t, *J*= 7.2 Hz, 1H), $H_{i}$ 7.52 (d, *J*= 8.8 Hz, 1H). ^13^C-NMR (100 MHz, DMSO-d_6_) δ (ppm): C=O ketone 192, 24 aromatic Carbon: 162.5, 160.5, 154.2, 150.7, 148.4, 146.5, 144.0, 141.9, 139.9, 139.7, 137.6, 135.9, 135.4, 133.1, 131.3, 129.1, 128.7, 128.3, 125.6, 125.0, 124.6, 124.5, 123.6, 122.9, and 121.9.

**IR (4a)**

**^1^H-NMR**

**^13^C-NMR**

**Synthesis of 7-(3-Nitro Phenyl) 8,13-Dihidro-H7-Benzo [h] Indeno [1, 2 b] quinoline-8-on [4a´]**

In double necked round-balloon equipped with a condenser, a mixture consisting of 1, 3-indandeion (0.146 g, 1 mmol), 3-Nitro Benzaldehide (0.151 g, 1 mmol), and 1-Naphtyle Amine (0.143 g, 1 mmol) in presence of the ([cmdmim]I) catalyst (10% molar), water solvent (10.0 ml) was heated for15 min at reflux conditions. The progress of the reaction was followed by the TLC chromatography (eluting with a mixture of Petroleum Ether, Ethyl Acetate (3: 10)). After cooling and solvent evaporation, the mixture of reaction was dissolved in Ethanol and chloroform (40: 20 ml) and then, it was purified and dried. The red powder of the 4a´ (0.378 g) was produced in 94% yield and 275-277°C. The 4b´ compound has been synthesized from scheme S2, which its physical property is given in Table S6. On the other hands, comparison proposed method for synthesis of the 4a is given in Table S7.

**Table S6.** Synthesis of 7-Aryl 8,13-Dihidro-H7-Benzo [h] Indeno [1, 2 b] quinoline-8-ons [4a´ and 4b´] compounds.

| **Product** | **Color of powder** | **Melting point (°C)** | **Time (min)** | **Efficiency (%)** |
| --- | --- | --- | --- | --- |
| 4a´ | Red | 275.0-277.0 | 12.0 | 94.0 |
| 4b´ | Red | 285.0-287.0 | 12.0 | 92.0 |

**Table S7.** Comparison of proposed method for synthesis of 7-Aryl 8,13-Dihidro-H7-Benzo [h] Indeno [1, 2 b] quinoline-8-ons [4a´] compound in presence of (cmdmim)I ionic liquid catalyst with other used method.

| **catalyst** | **Value (%mol)** | **Solvent** | **Temperature (°C)** | **Time (min)** | **Efficiency (%)** | **Ref** |
| --- | --- | --- | --- | --- | --- | --- |
| (cmdmim)I | 10.0 | Water | Reflux | 12.0 | 92.0 | Proposed |
| Poly (2-Acryl Amido-2-Methyl Propane Sulphonic Acid) | 15.0 | Aceto Nitryl | Reflux | 120.0 | 92.0 | (^3^) |

Red powder of the 4a´ compound synthesized from interaction of between 1, 3 Indandion (1mmol), 3 Nitro Phenyl Benzaldehyde (1mmol) and 1 Naphtil Amine (1mmol) in presence of the [cmdmim]I ionic liquid catalyst, water solvent at Argon atmospheric, and 12 min with 94.0% efficiency, see scheme S2.

**Scheme S2.** Synthesis of 7-Aryl 8,13-Dihidro-H7-Benzo [h] Indeno [1, 2 b] quinoline-8-on [4a´] compound.

The **4a´** compound was confirmed using the IR, ^1^H-NMR, and ^13^C-NMR stereoscopies.

IR (KBr): 3423, 3283 (N-H stretch), 3091 (C-H stretch, aromatic), 2927, 2864 (aliphatic C-H stretch), 1725 (C=O stretch), 1582, 1463 (C-C stretch, aromatic), 1522, 1341 (NO_2_ stretch), 858, 809, 757, 714 (aromatic C-H out of plane bending) cm^-1^. ^1^H NMR (300 MHz, DMSO-d_6_): δ; 10.30 (s, 1H, He), 8.77 (d, *J* = 8.4 Hz, 1H, Hf), 8.15 (d, *J*= 6.6 Hz, 1H, Hn or Hi), 8.14 (s, 1H, Hm), 8.03 (d, *J* = 8.1 Hz, 1H, Hi or Hn), 7.91 (d, *J* = 7.8, 1H, Hd), 7.78-7.69 (m, 2H, Ho, Hp), 7.63-7.27 (m, 7H, Ha, Hb, Hc, Hg, Hh, Hj, Hk), 5.56 (s, 1H, Hl) ppm.^13^C NMR (100 MHz, DMSO-d_6_): δ; 190.7 (C=O), 156.4, 149.6, 148.3, 136.9, 135.2, 134.4, 133.3, 131.9, 131.6, 130.8, 130.6, 128.8, 127.1, 127.0, 126.9, 125.0, 123.8, 122.6, 122.4, 121.9, 121.4, 121.0, 120.6, 105.5, 32.0 ppm.

**IR (KBr): stretching and bending frequencies**: N—H (3423 and 3283 cm^-1^), aromatic C—H (3091 cm^-1^), aliphatic C—H (2958, 2927, and 2863 cm^-1^), C=O (1725 cm^-1^), aromatic C—C (1528 and 1463 cm^-1^), $\mathrm{NO}_{2}$ (1522 and 1341 cm^-1^) and N—H (1660 cm^-1^), aromatic C—H out of plan (858, 809, 757, and 714 cm^-1^), respectively.

**^1^H-NMR**

**^1^H-NMR (300 MHz, DMSO-d_6_)** δ (ppm): $H_{e}$ 10.30 (s, *broad*), $H_{f}$ 8.77 (d, *J*= 11.2 Hz, 1H), $H_{n}$ or $H_{i}$8.15 (d, *J*= 8.8 Hz, 1H),$H_{m}$ 8.14 (s, 1H), $H_{i}$ or $H_{n}$ 8.03 (d, *J*= 10.8 Hz, 1H), $H_{d}$ 7.91 (d, *J*= 10.4 Hz, 1H), $H_{O}$ and $H_{p}$7.69-7.78 (m, 2H), $H_{a}$, $H_{b}$, $H_{c}$, $H_{g}$, $H_{h}$, $H_{j}$ and $H_{k}$ 7.27-7.63 (m, 7H), $H_{l}$ 5.56 (s, 1H).

**^13^C-NMR**

**^13^C-NMR (100 MHz, DMSO-d_6_, 26°C)** δ (ppm): C=O ketone 190.7, 25 aromatic Carbon: 156.4, 149.6, 148.3, 136.9, 135.2, 134.4, 133.3, 131.9, 131.6, 130.8, 130.6, 128.9, 128.8, 127.1, 127.0, 126.9, 125.0, 123.8, 122.6, 122.4, 121.9, 121.4, 121.0, 120.6, 105.5and 32.0, see Figure 6.

The IR, ^1^H-NMR, and ^13^C-NMR stereoscopies of other fused quinolines are given in Appendix.

**Theoretical calculations**

**Quantum mechanics (QM) study**

A theoretical study can help to understand and investigate unknown mechanistic aspects and the best carrier of synthesis drug molecules. Geometry optimization and frequency test have been carried out using ωB97-XD/6-31g* level of theory. These calculations show that all structures are in real minima, see Figure S1.

In the armchair SWBNNT (n, n; length, diameter) (length and diameter are in Angstrom (Å)), “n”, length and diameters indexes are 6, 15 and 8Å, respectively. The 78 Boron, 78 Nitrogen, and 24 Hydrogen atoms of this nanotube has been modeled based on cluster approach (Nanzi 2002). Range of atomic charges in nanotube is -0.570 to +0.570e with 960e in the singlet multiplicities. Besides, there are quadru and hexa-deca pole moments without any dipole moment.

**Electronic properties of isolated structures**

Investigating active sites and predicting strength of interactions in the 4a, derivatives and SWBNNT is carried out using molecular electrostatic potential (MEP) maps ^4^. The yellow, blue, and green colors elucidate the most negative, positive, and zero electrostatic potential, respectively, as shown in Figure S1.

The highest occupied molecular orbitals (HOMO) and lowest unoccupied molecular orbitals (LOMO) of the **4a** molecule and SWBNNT have been plotted due to more understanding of their interaction, see Figure S2. According to this figure, it should be expected that more charge transfer is carried out from SWBNNT to **4a** molecule due to lower the HOMO_SWBNNT_-LUMO_4a_ gap than others. This issue confirms by 125 kcal mol^-1^ in total charge transfer from SWBNNT to the 4a molecule than 89 kcal mol^-1^ in vice versa.


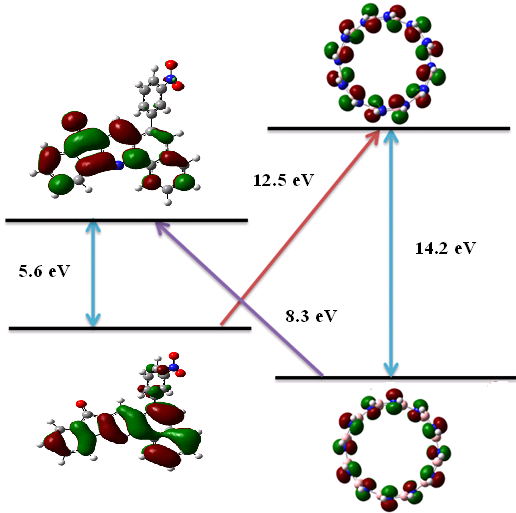


**Figure S2.** Calculated HOMO, LUMO orbitals of the 4a molecule and SWBNNT in ωB97-XD/6-31g(d) level of theory.

**Interaction between SWBNNT and 4a**

In order to investigating the best favorable sites and molecule tendency of the 4a and derivative molecules in their interactions, global and local reactivity calculations have been carried out, see Tables S8. According to this table, more and less negative value of the ***µ*** is related to the 4a, 4b, 4c, and 4i molecules and the SWBNNT where indicates that they accept and donate electron conveniently. This issue is confirmed by low the HOMO_SWBNNT_-LUMO_4x_, low the ***η***, and high the ***S***, see Table 10. On the other hands, high the ***ω*** show that floating of electron between SWBNNT with 4a and 4b molecules is carried out conveniently.

**Table S8.** Global reactivity indexes (in electron volt) of the 4a and derivative molecules and adsorption energy (in kJ mol^-1^) at selected level of theory.

| **molecule** | **IE** | **EA** | ***µ*** | ***η*** | ***S*** | ***ω*** | ***∆*H_SWBNNT_-L_4x_** | ***E_ads_*** |
| --- | --- | --- | --- | --- | --- | --- | --- | --- |
| **4a** | 6.05 | 3.05 | -4.55 | 1.50 | 0.67 | 6.91 | 4.78 | -87.9 |
| **4b** | 6.06 | 3.06 | -4.56 | 1.50 | 0.67 | 6.94 | 4.77 | -72.8 |
| **4c** | 6.05 | 3.06 | -4.55 | 1.50 | 0.67 | 6.93 | 4.99 | -32.6 |
| **4d** | 6.08 | 2.93 | -4.51 | 1.58 | 0.64 | 6.45 | 4.93 | -14.4 |
| **4e** | 5.82 | 3.01 | -4.42 | 1.41 | 0.71 | 6.93 | 5.03 | -23.2 |
| **4f** | 5.87 | 2.96 | -4.42 | 1.45 | 0.69 | 6.72 | 5.03 | -15.7 |
| **4g** | 5.89 | 2.96 | -4.43 | 1.47 | 0.68 | 6.67 | 5.08 | -12.6 |
| **4h** | 6.07 | 2.95 | -4.51 | 1.56 | 0.64 | 6.51 | 4.90 | -10.7 |
| **4i** | 6.14 | 2.96 | -4.55 | 1.59 | 0.63 | 6.52 | 4.89 | -21.3 |
| **4j** | 6.07 | 2.95 | -4.51 | 1.56 | 0.64 | 6.51 | 4.90 | -10.7 |
| **4k** | 6.09 | 2.95 | -4.52 | 1.57 | 0.64 | 6.49 | 4.90 | -13.2 |
| **4l** | 6.10 | 2.95 | -4.53 | 1.58 | 0.64 | 6.51 | 4.97 | -16.3 |
| **4** | 6.04 | 2.92 | -4.48 | 1.56 | 0.64 | 6.44 | 4.99 | -10.7 |
| **SWBNNT** | 6.47 | 0.07 | -3.27 | 3.20 | 0.31 | 1.67 | - | - |

***∆*H_SWBNNT_-L_4x_:** Gap of the SWBNNT HOMOs and 4a and derivatives LUMOs.

With investigating of their role, they were interacted with each other in sutable orientation, see Figure S3. Energy of stable configurations was considered to calculate of adsorption energy ($E_{ads}$). Based on the more negative value of the E*_ads_*, 4a and 4b complex has adsorption strength and stable configuration than others, as shown in Table S8.

Density of state (DOS) analysis has been carried out due to better understanding of these interactions. Total DOS of the 4a molecule, SWBNNT, and in/outside complexes are given in Figure S1. The reduction of energy gaps in complexes can be attributed to better interactions. These interactions may influence on electron excitation and excited states spectrum.

| **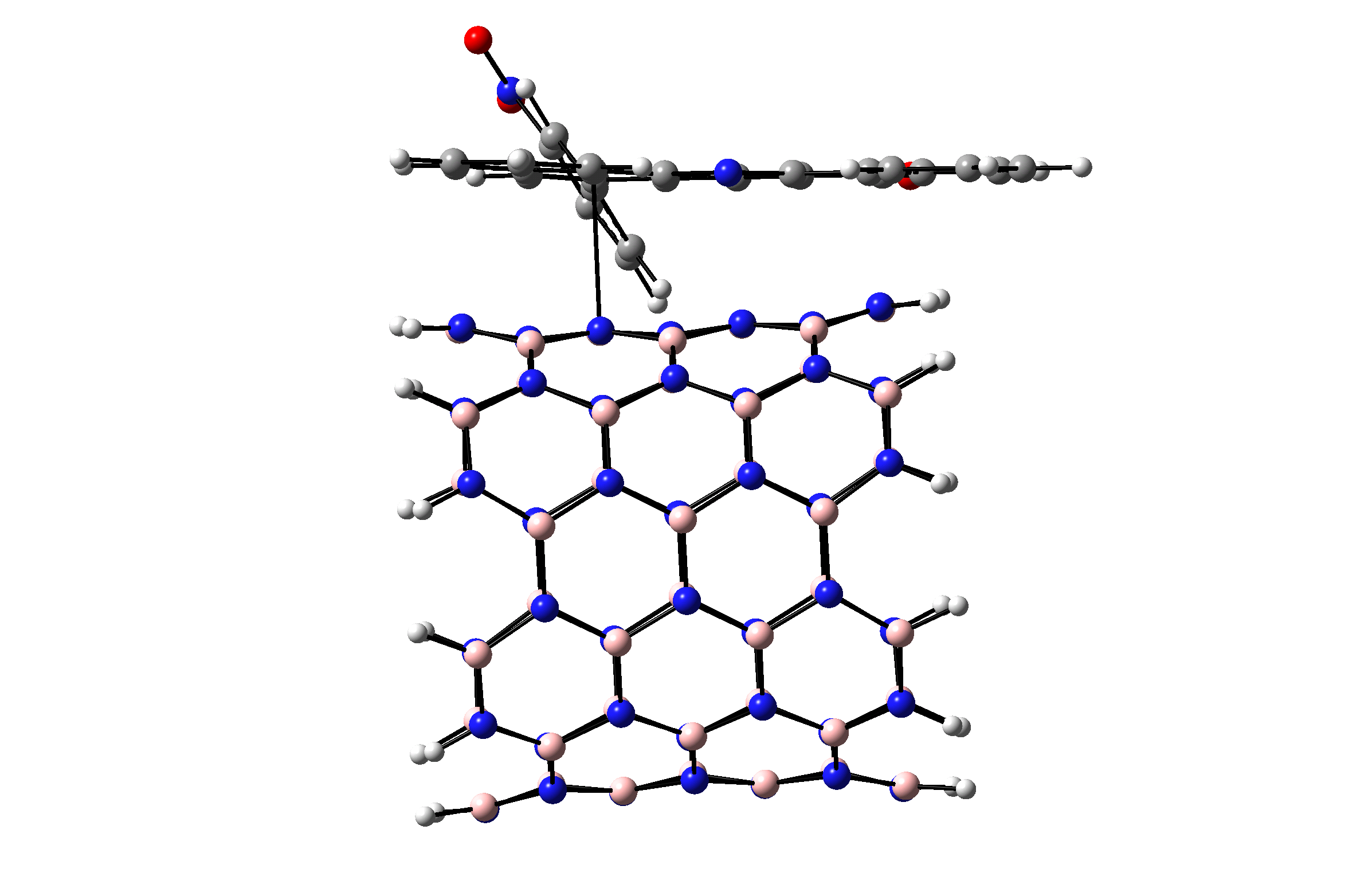**  **(4a)** | **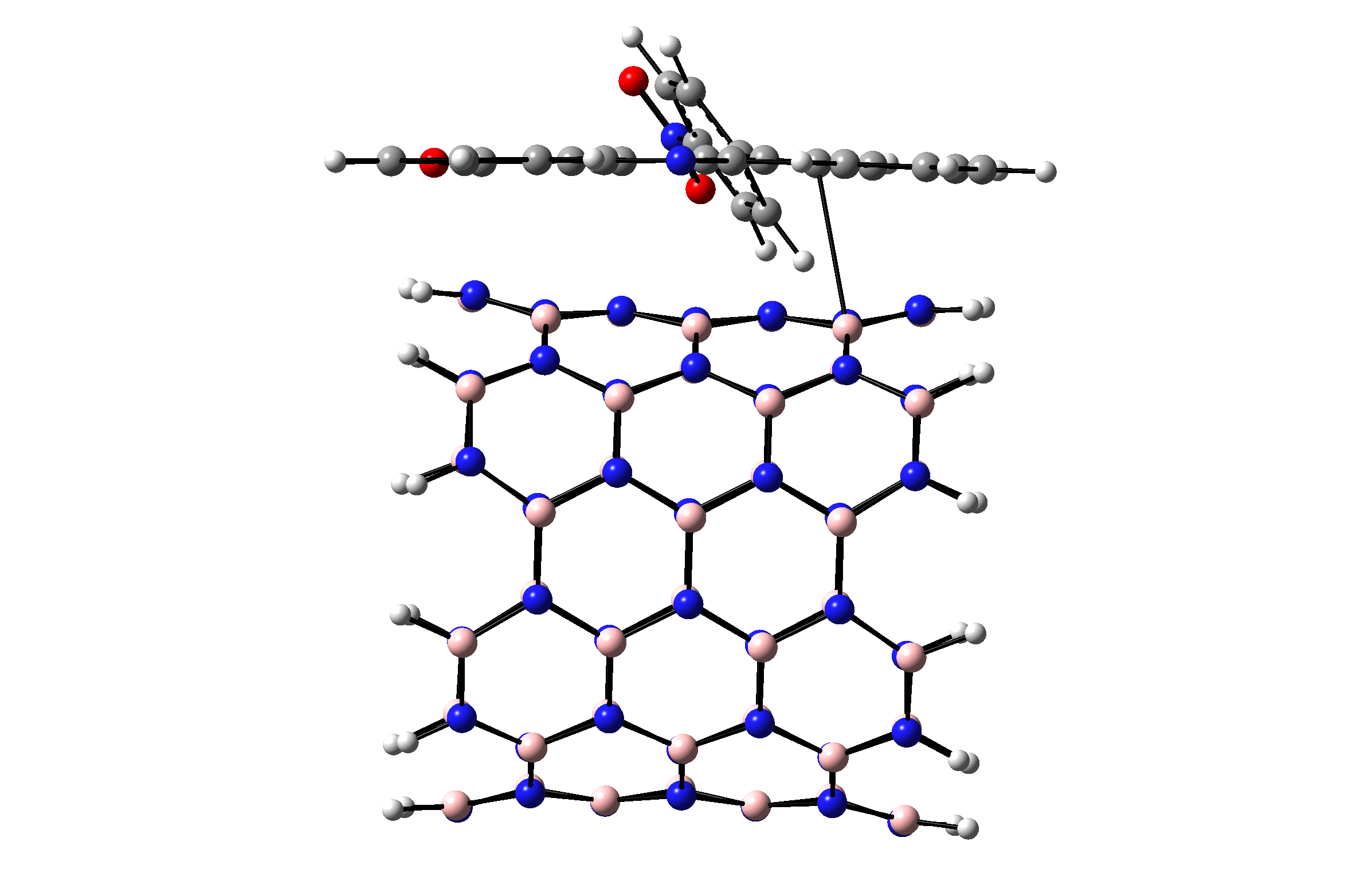**  **(4b)** |
| --- | --- |

**Figure S3.** Suitable geometry of interaction between 4a and 4b with SWBNNT in ωB97-XD/6-31g(d) level of theory.

**
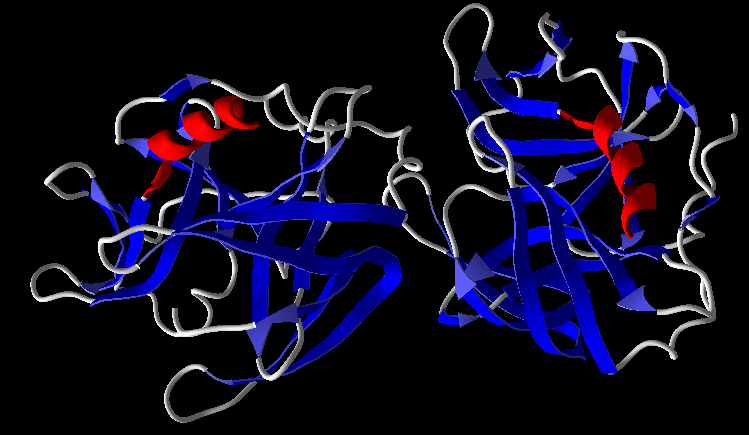
**

**Figure S4.** Three dimensional of Hepatitis A virus in secondary structure form with 1HAV PDB ID.

**1**

| **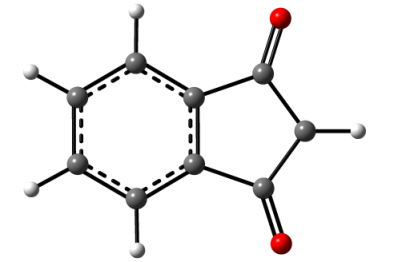** | **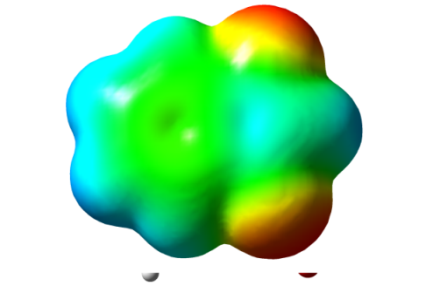** |
| --- | --- |
| **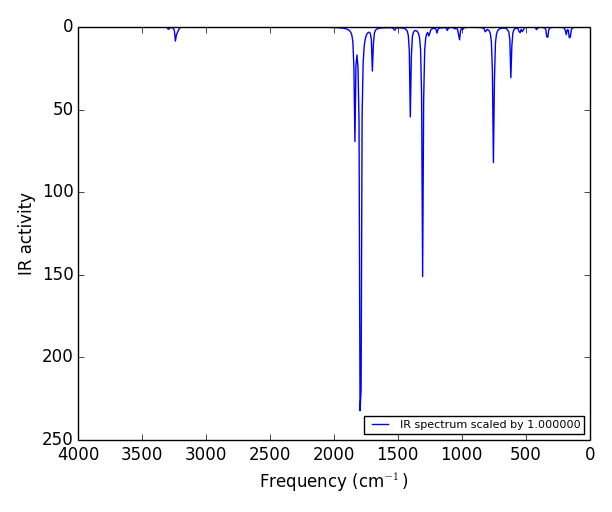** | |
| **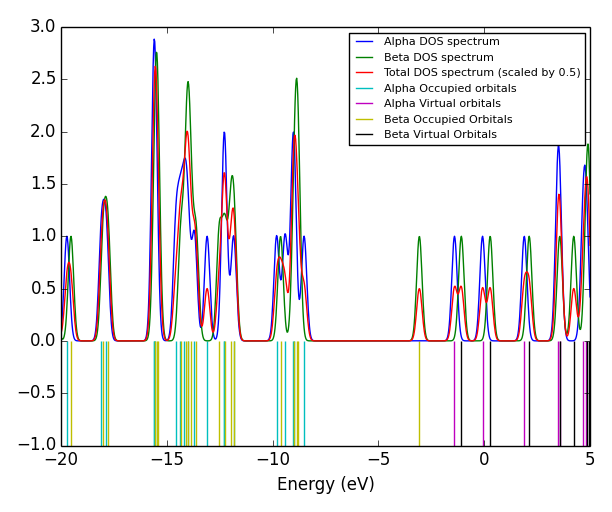** | |

**2 2Cl**

| **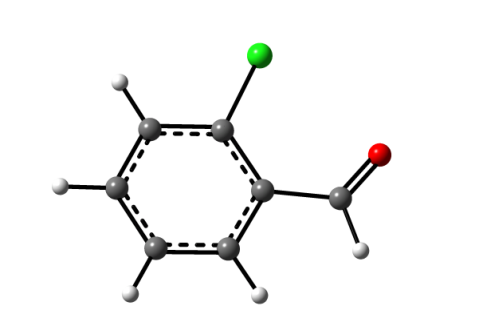** | **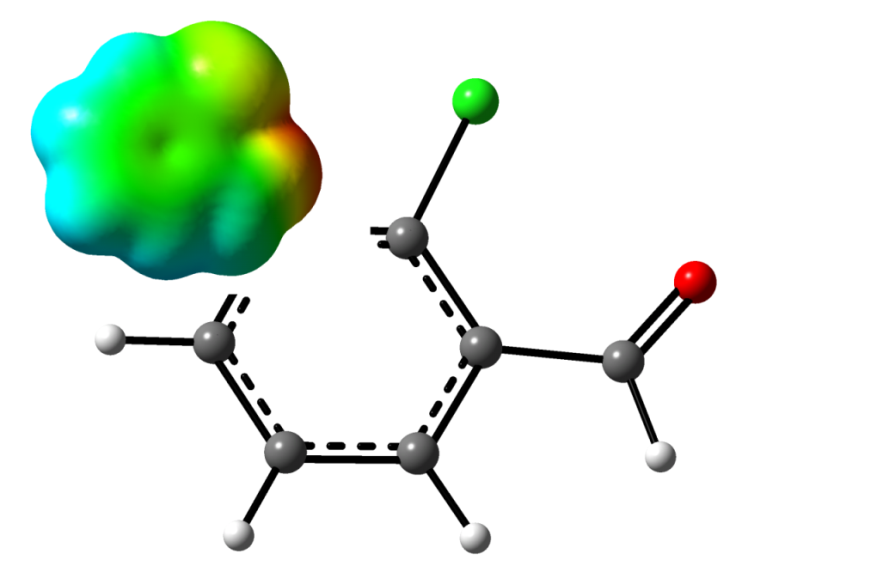** |
| --- | --- |
| **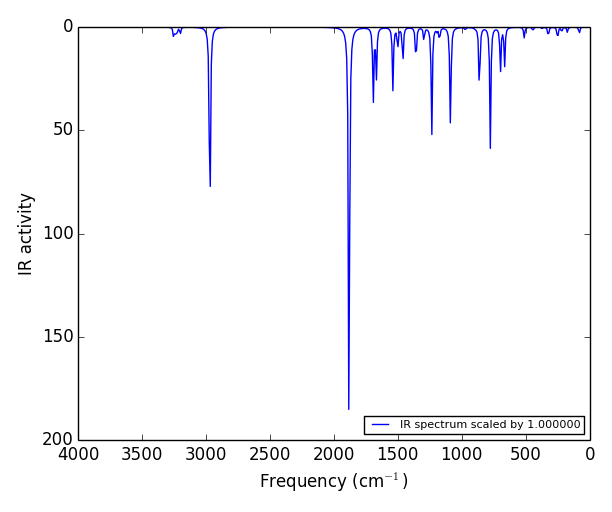** | |
| **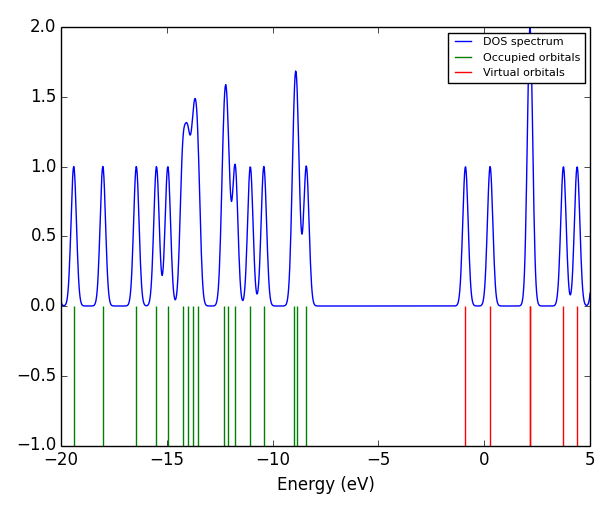** | |

**2 2NO_2_**

| **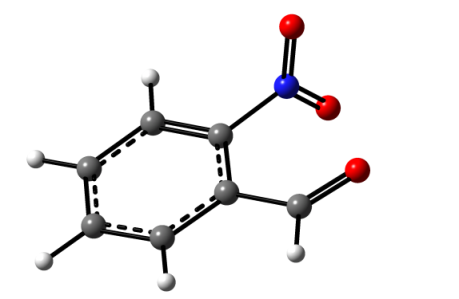** | **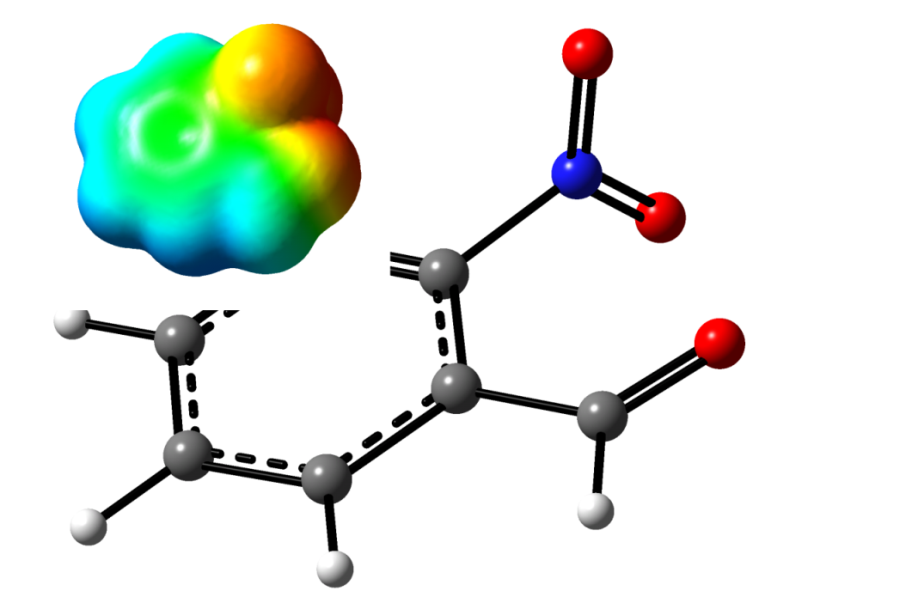** |
| --- | --- |
| **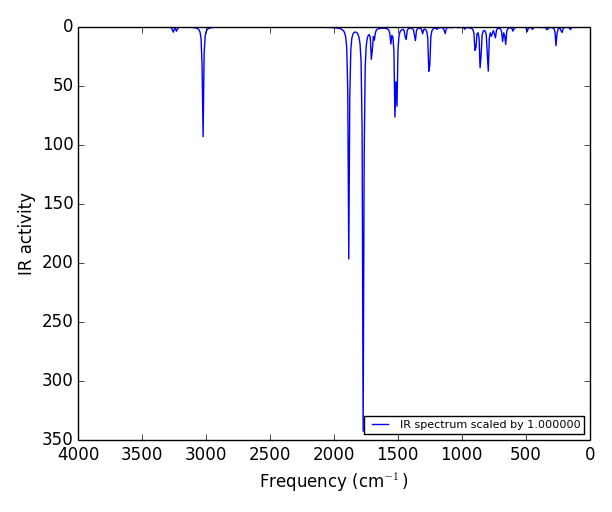** | |
| **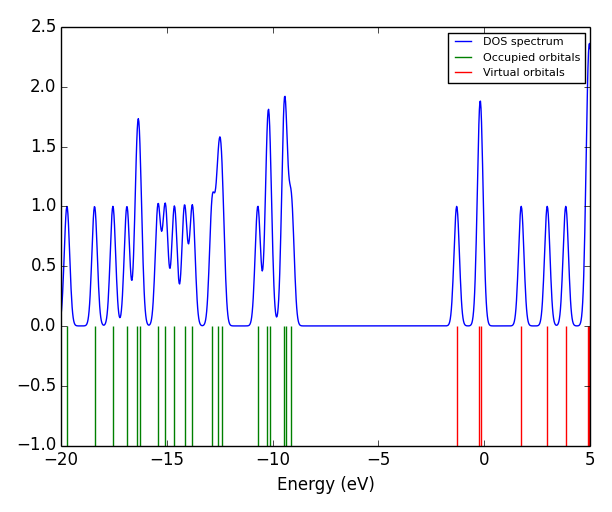** | |

**2 2OMe**

| **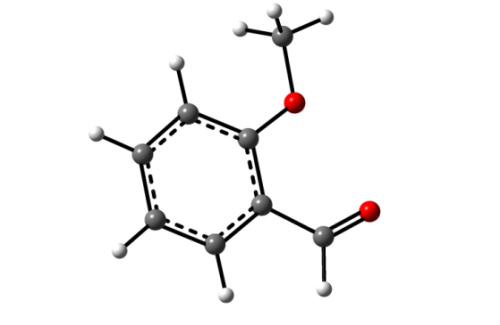** | **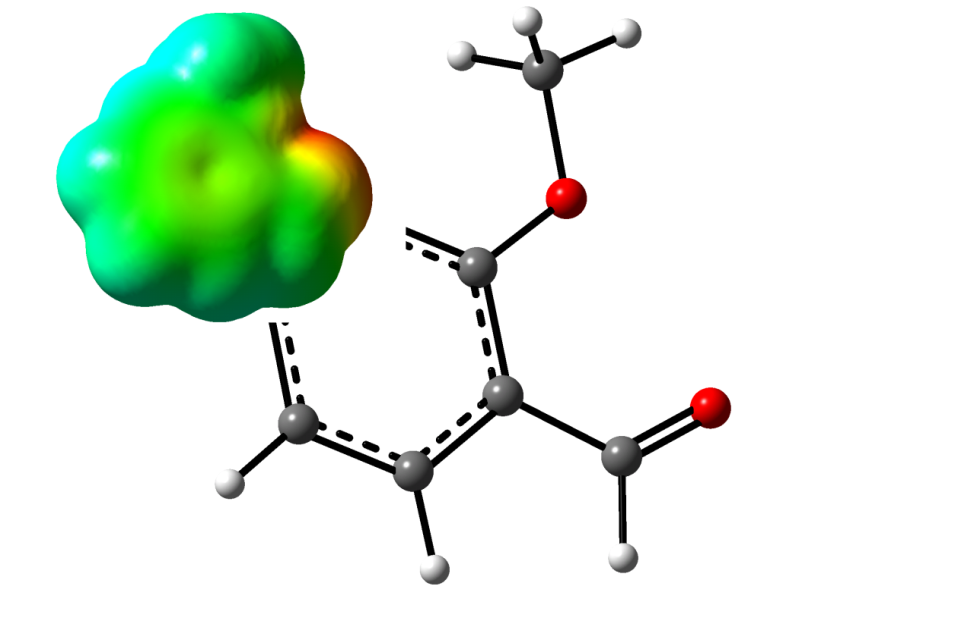** |
| --- | --- |
| **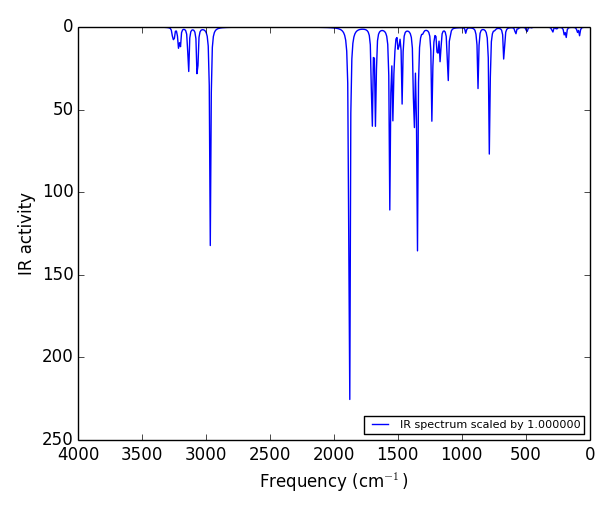** | |
| **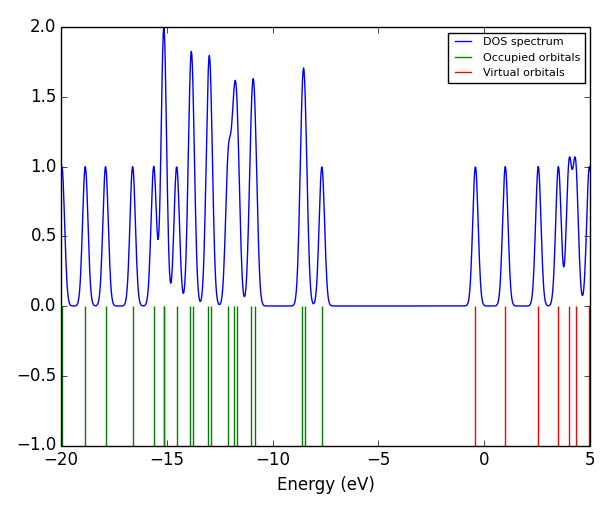** | |

**2 3Cl**

| **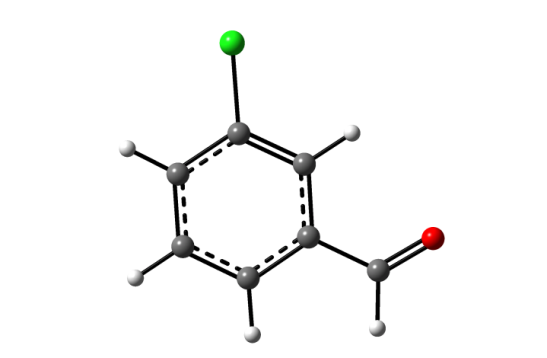** | **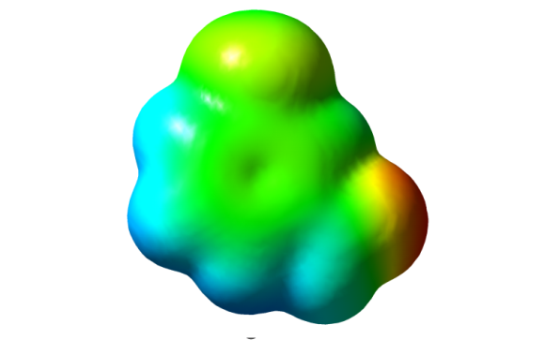** |
| --- | --- |
| **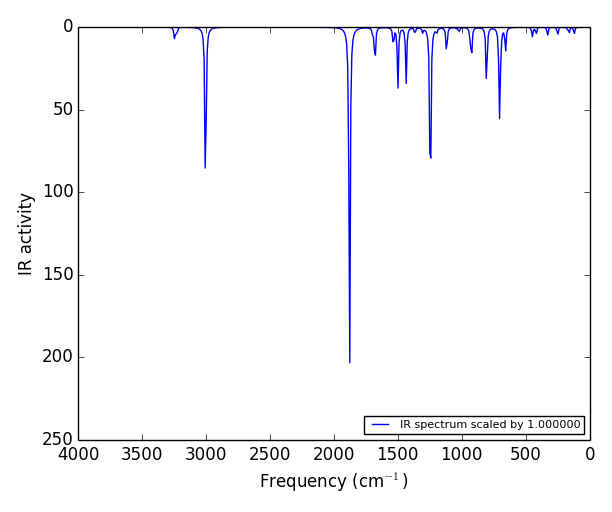** | |
| **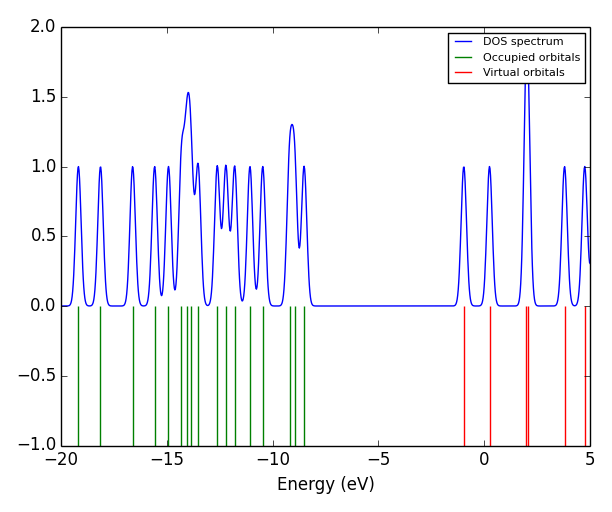** | |

**2 3F**

| **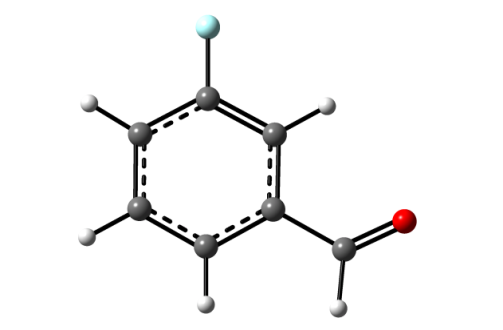** | **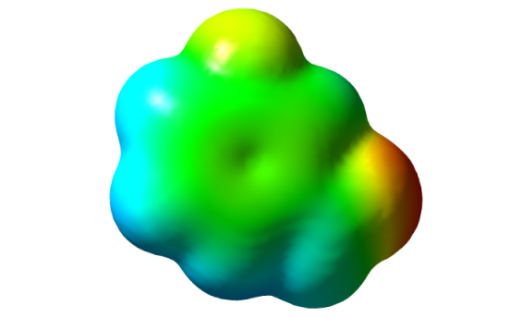** |
| --- | --- |
| **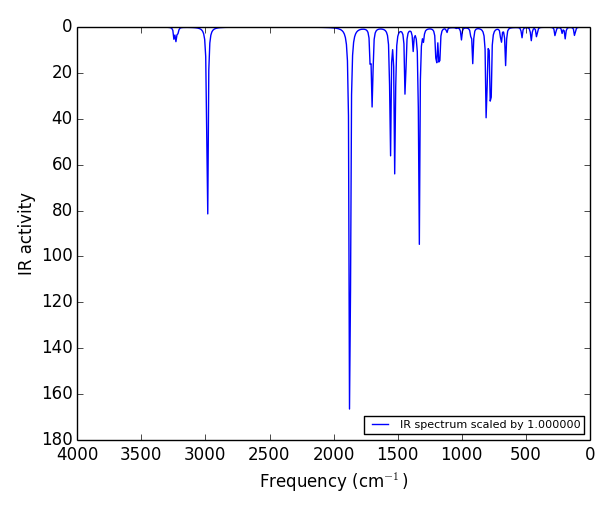** | |
| **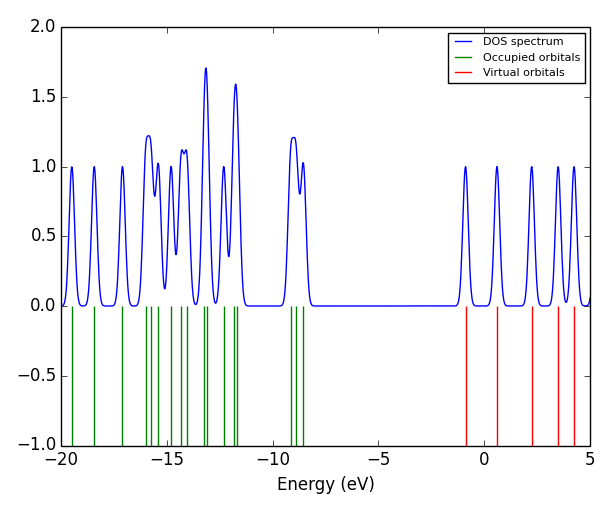** | |

**2 3NO2**

| **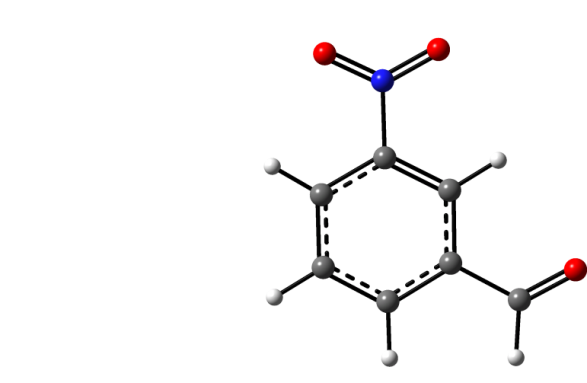** | **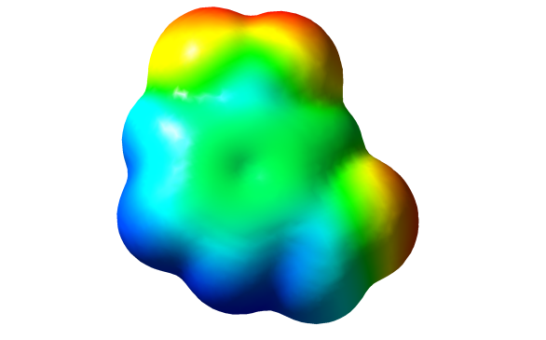** |
| --- | --- |
| **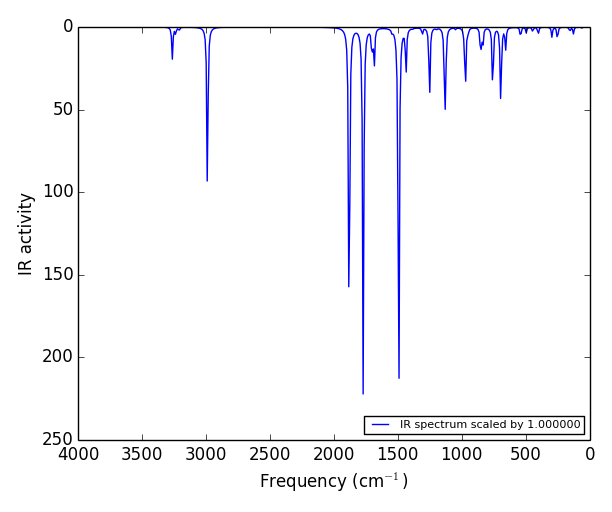** | |
| **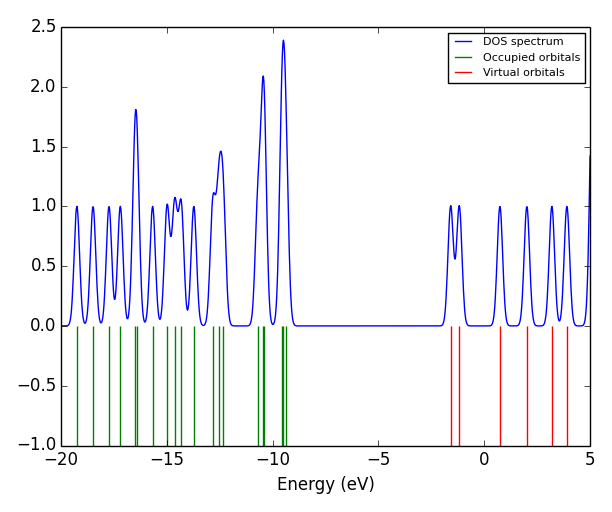** | |

**2 4Br**

| **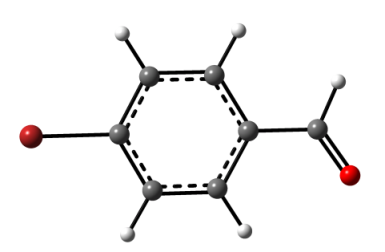** | **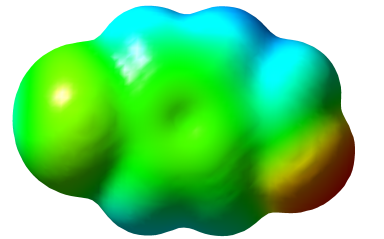** |
| --- | --- |
| **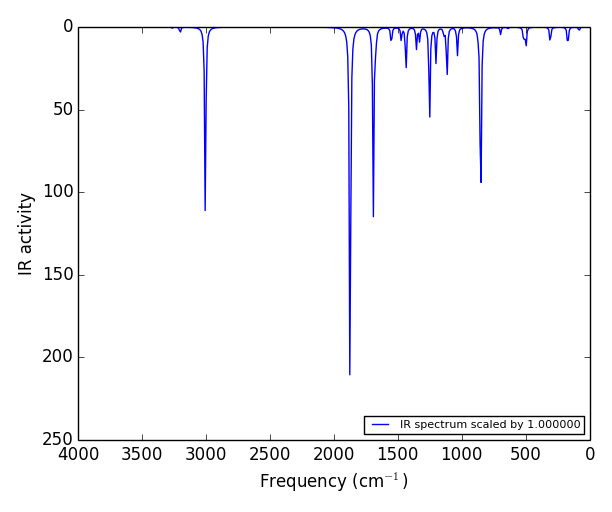** | |
| **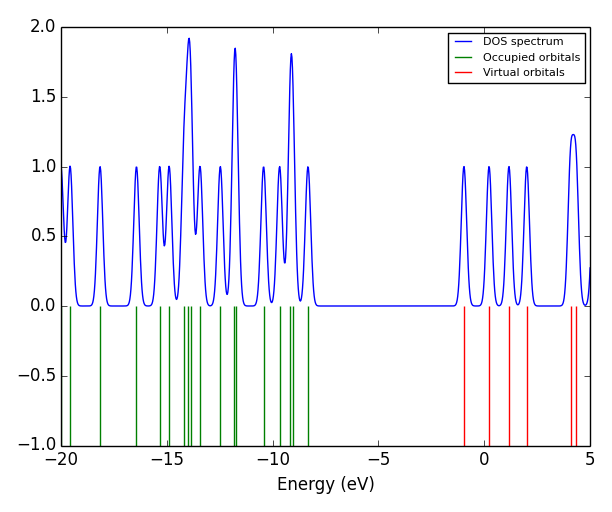** | |

**2 4(Cl)2**

| **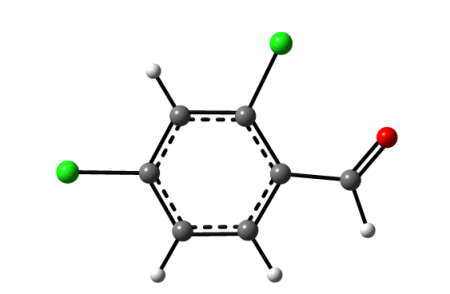** | **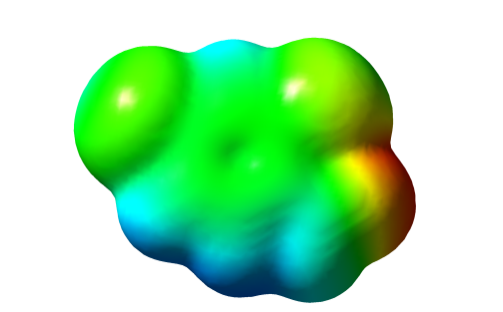** |
| --- | --- |
| **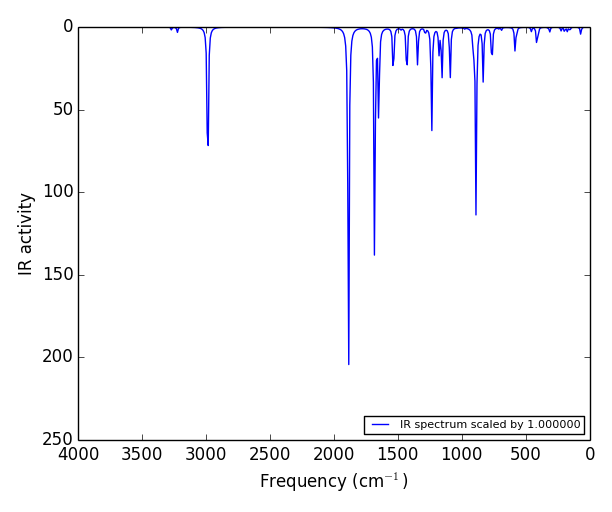** | |
| **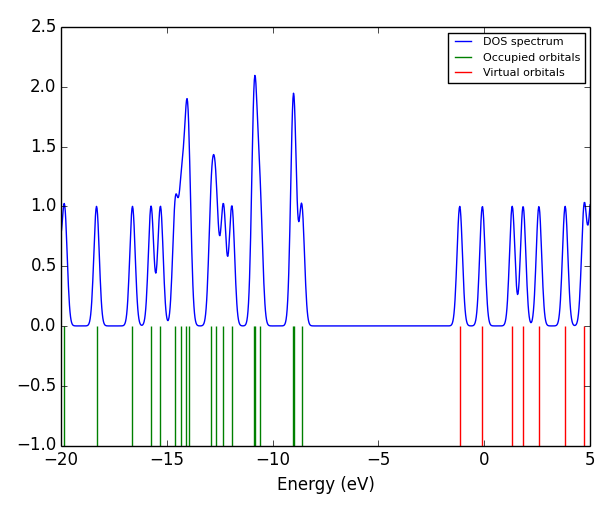** | |

**2 4Cl**

| **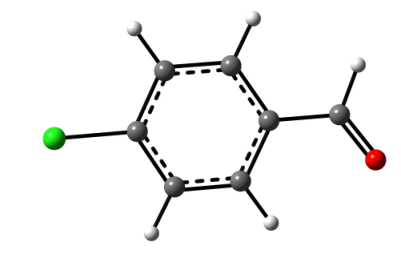** | **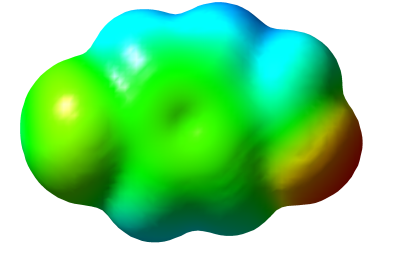** |
| --- | --- |
| **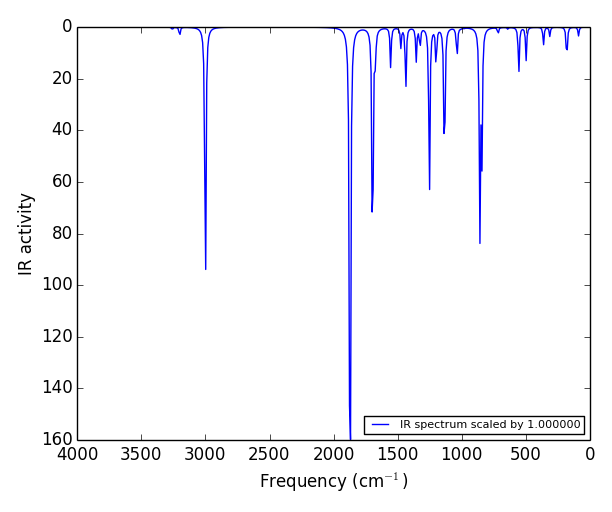** | |
| **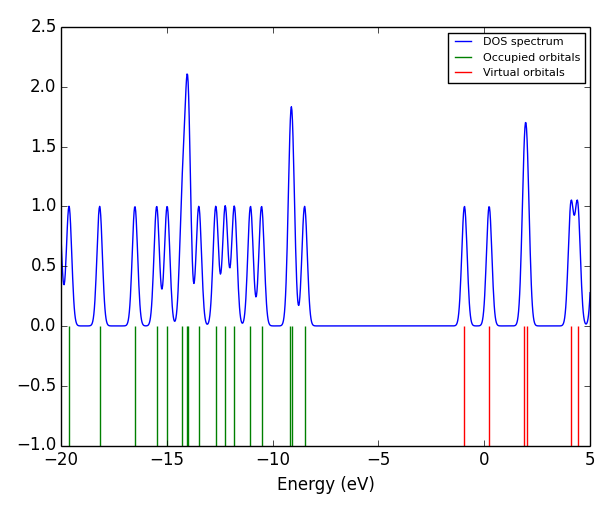** | |

**2 4NO_2_**

| **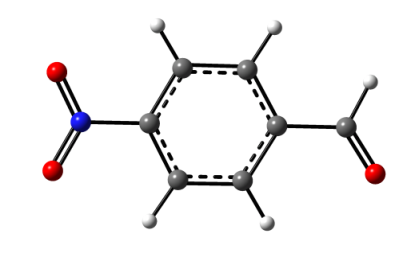** | **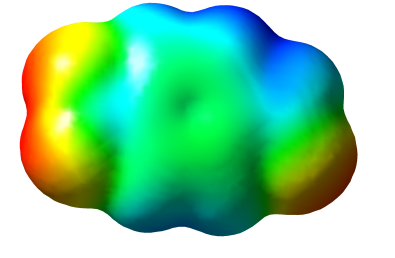** |
| --- | --- |
| **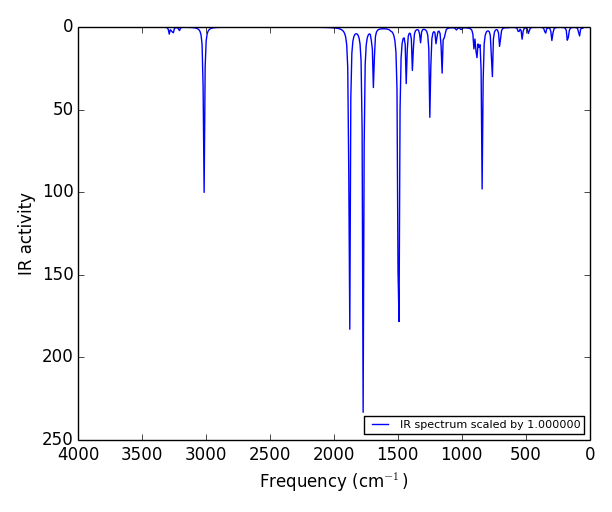** | |
| **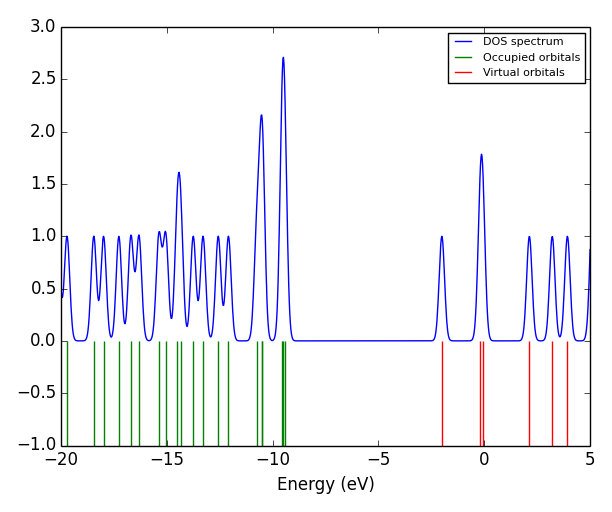** | |

**2 4OMe**

| **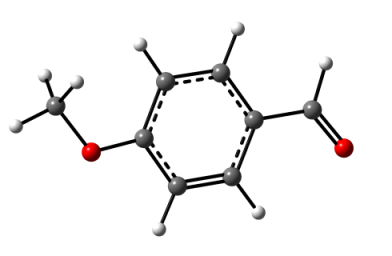** | **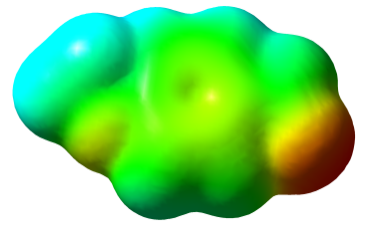** |
| --- | --- |
| **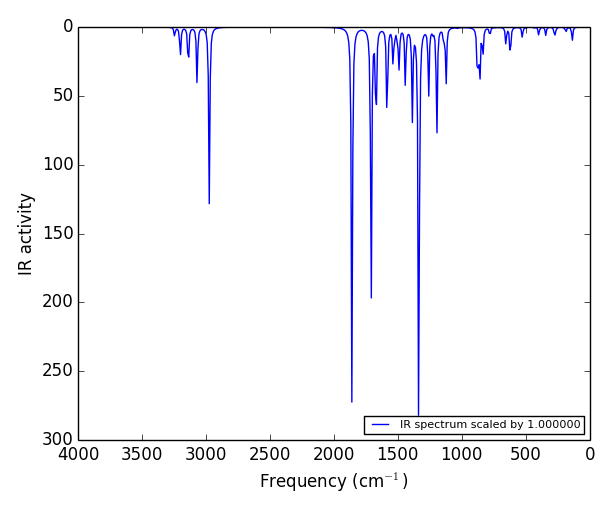** | |
| **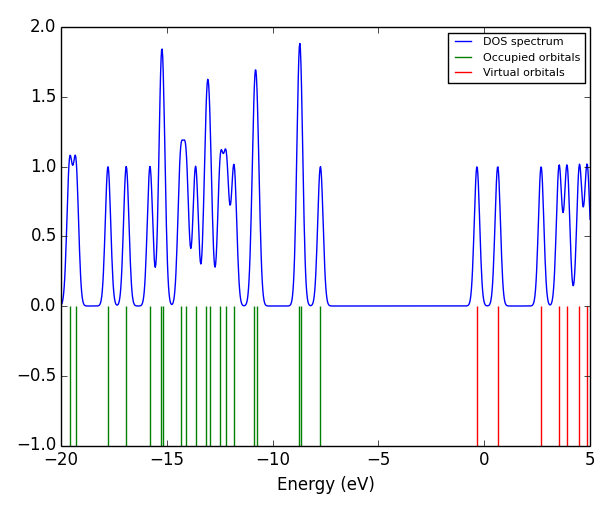** | |

**2 34(OMe)2**

| **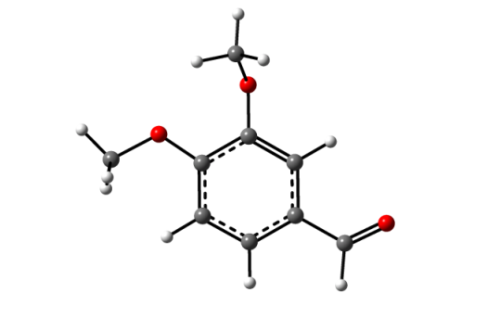** | **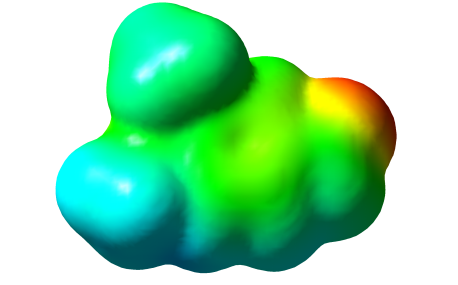** |
| --- | --- |
| **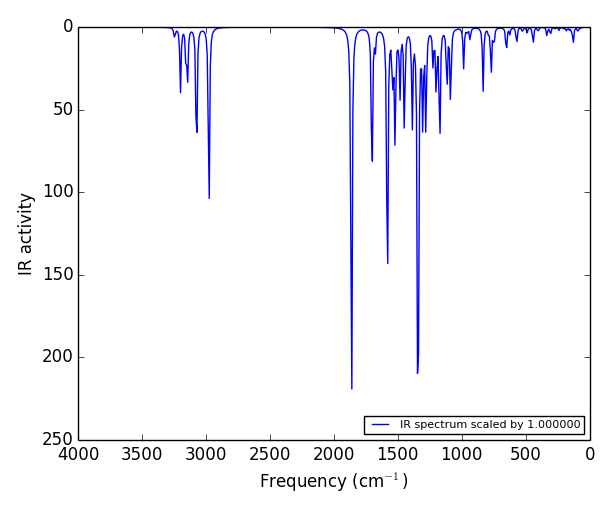** | |
| **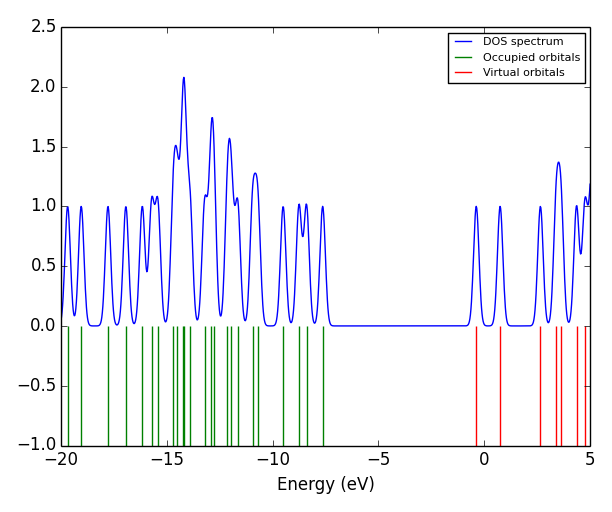** | |

**2**

| **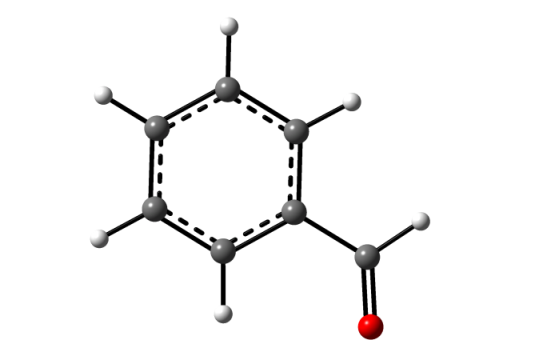** | **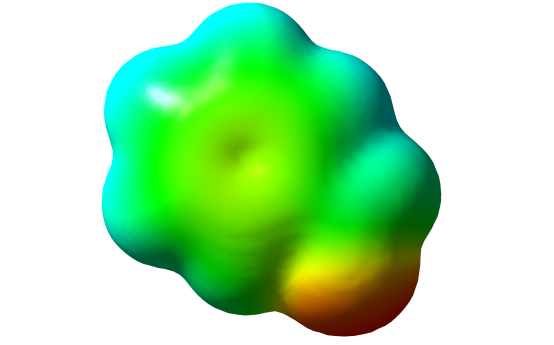** |
| --- | --- |
| **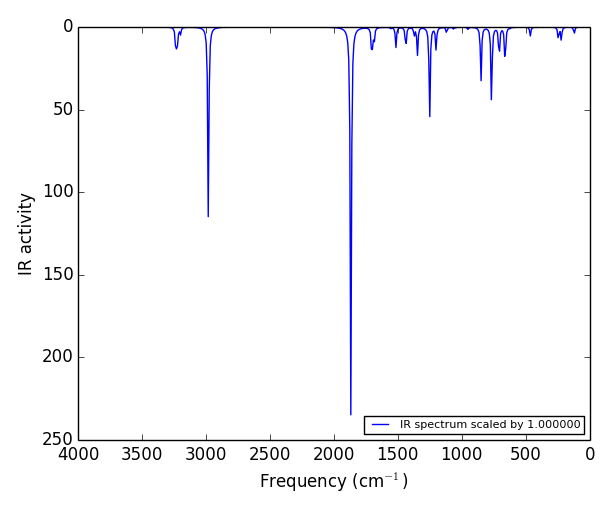** | |
| **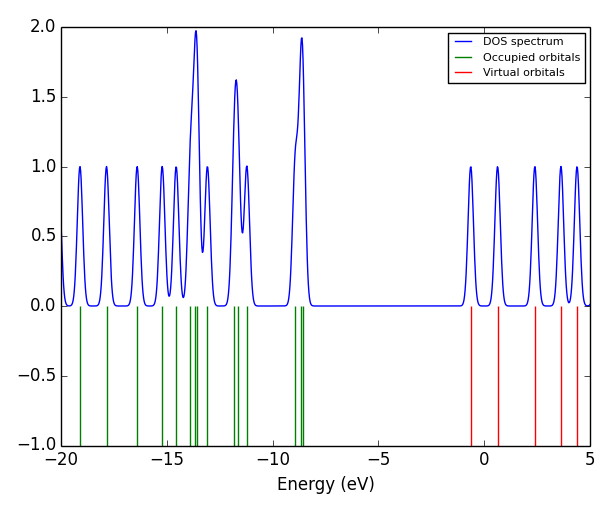** | |

**3**

| **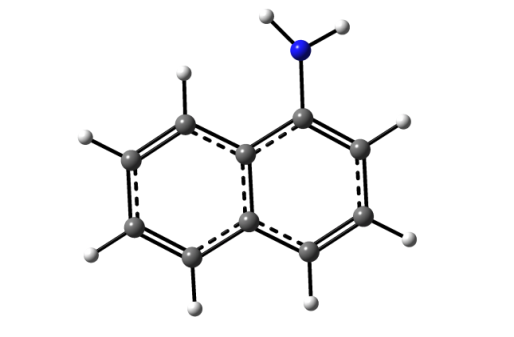** | **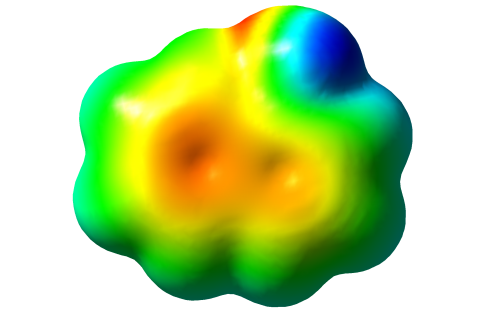** |
| --- | --- |
| **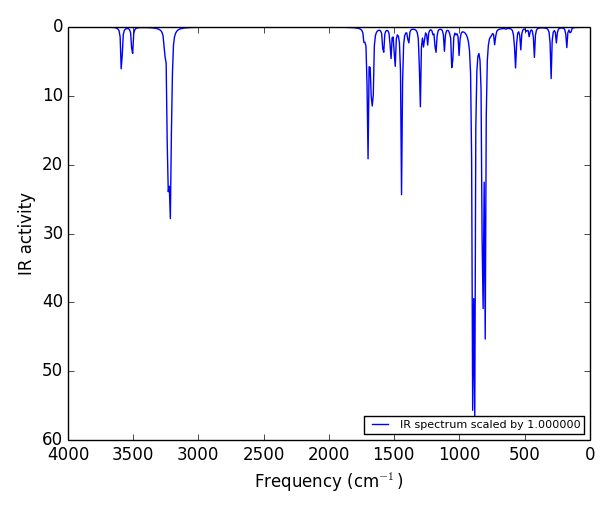** | |
| **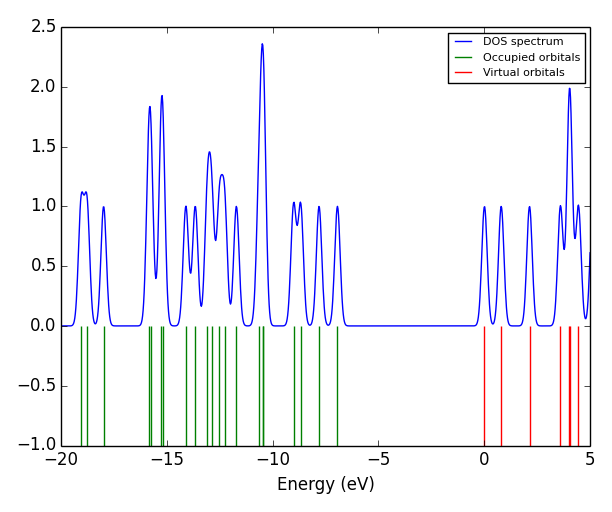** | |

**4**

| **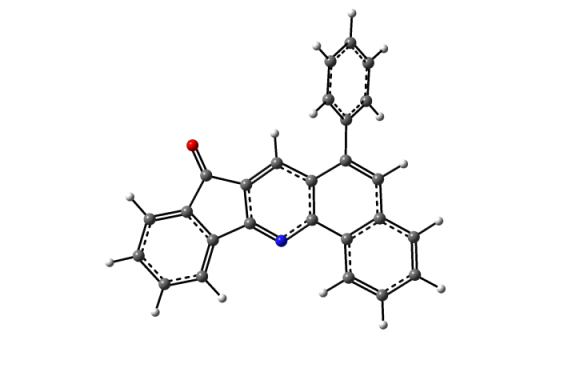** | **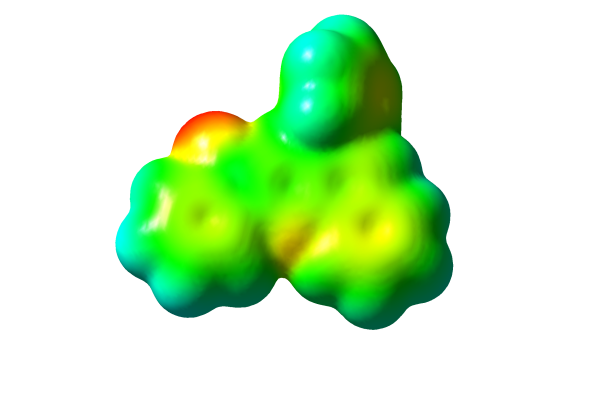** |
| --- | --- |
| **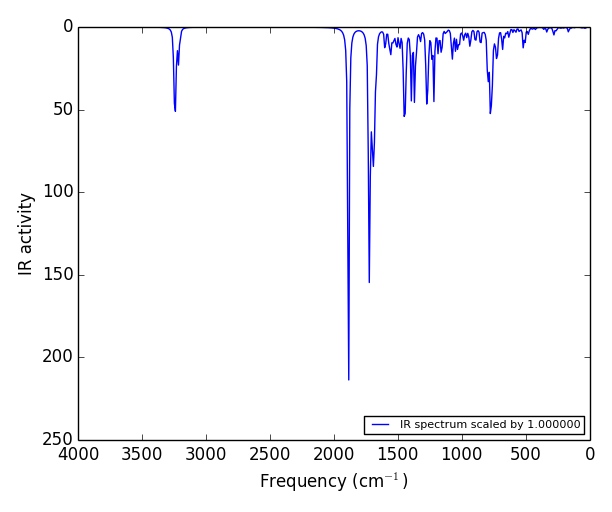** | |
| **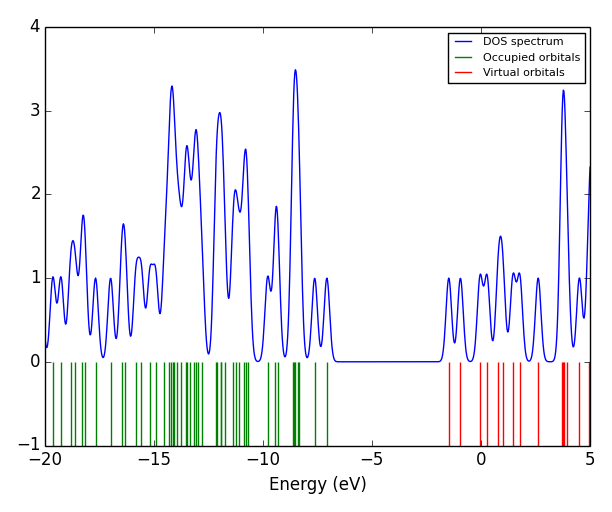** | |

**4a**

| **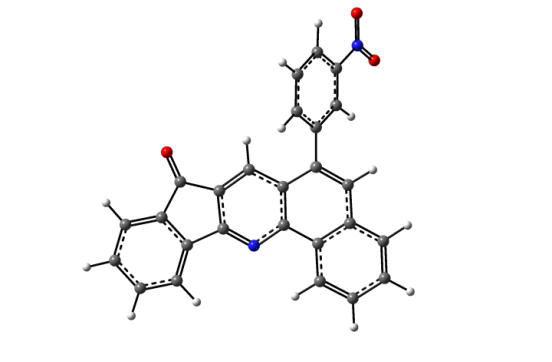** | **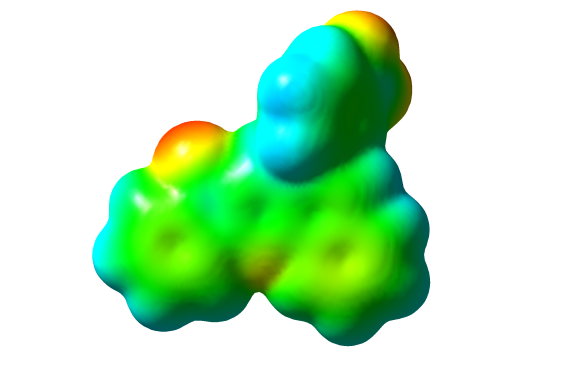** |
| --- | --- |
| **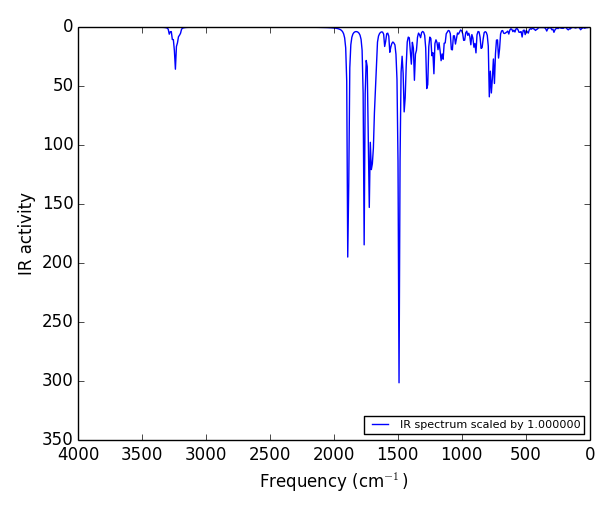** | |
| **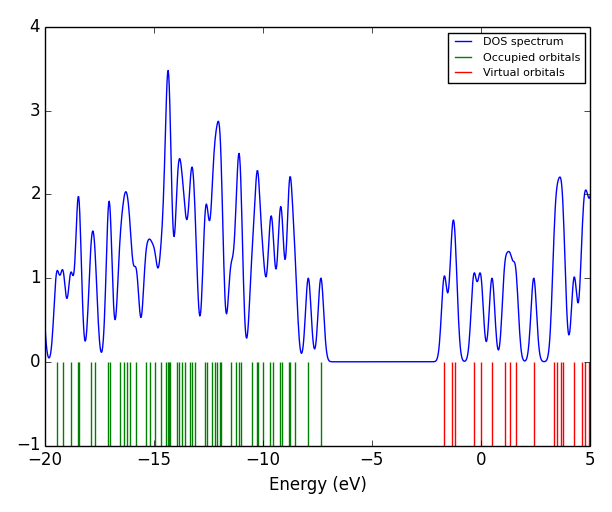** | |

**4b**

| **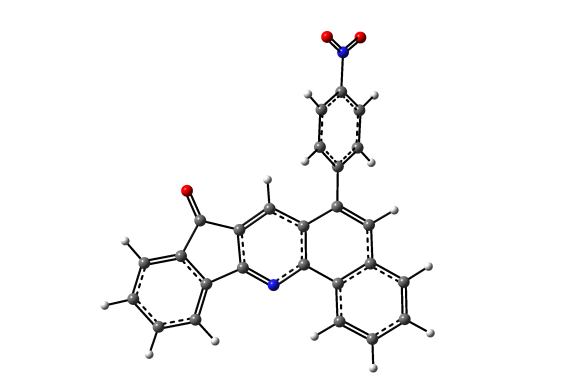** | **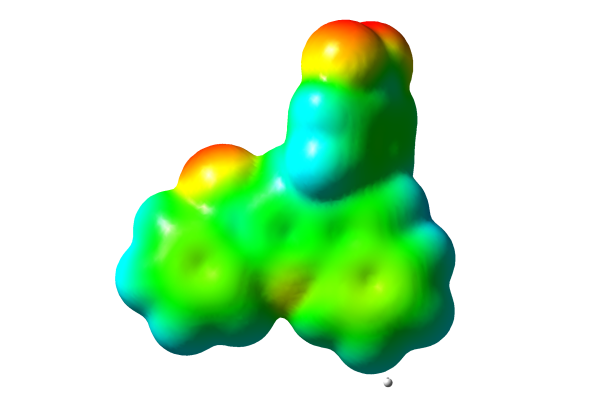** |
| --- | --- |
| **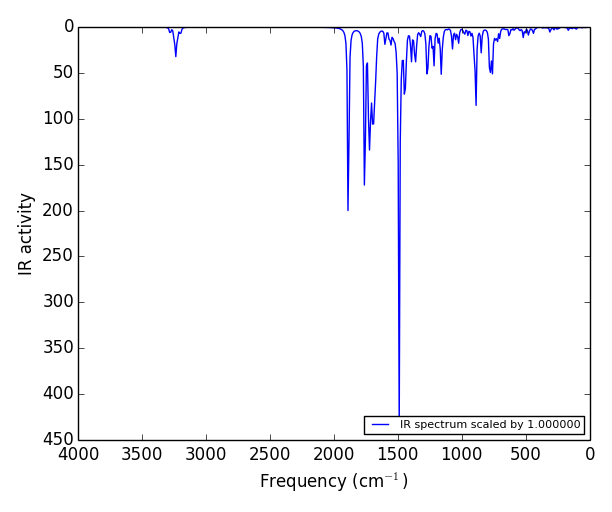** | |
| **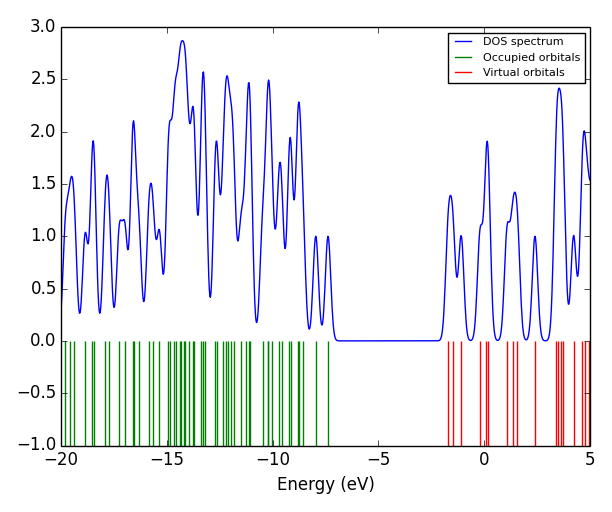** | |

**Figure S1.** Ground state structures, molecular electrostatic potential (MEP), theoretical infra-red (IR), and density of state (DOS) of the all structures in ωB97-XD/6-31g(d) level of theory, respectively.

**Appendix**

**IR (KBr):** 3055 (C-H stretch, aromatic), 1716 (C=O stretch), 1660 (C=N stretch), 1579, 1464 (C-C stretch, aromatic), 1513, 1340 (NO2 stretch), 1225 (C-CO-C bend), 856, 808, 755 (aromatic C-H out of plane bending) cm^-1^.

**4b´**

**IR (KBr):** 3322 (N-H stretch), 3057 (C-H stretch, aromatic), 2960, 2927, 2859 (aliphatic CH stretch), 1721 (C=O stretch), 1617, 1579, 1464 (C-C stretch, aromatic), 1526, 1344 (NO2 stretch), 862, 805, 757, 712 (aromatic C-H out of plane bending) cm^-1^.

**^1^H NMR (400 MHz, DMSO-d_6_):** δ; 10.28 (s, 1H, He), 8.78 (d, *J* = 8.4 Hz, 1H, Hf), 8.17-8.13 (m, 3H, Hn, Hi), 7.92 (d, *J* = 8.0 Hz, 1H, Hd), 7.72 (t, *J* = 7.6 Hz, 1H, Hc or Hb), 7.63-7.51 (m, 5H, Hc or Hb, Hh or Hg, Hj, Hm), 7.42 (t, *J* = 7.2 Hz, 1H, Hg or Hh), 7.31 (d, *J* = 7.2, 1H, Ha), 7.24 (d, *J* = 8.4, 1H, Hk) , 5.52 (s, 1H, Hl) ppm.

**^13^C NMR (100 MHz, DMSO-d_6_):** δ; 190.7 (C=O), 156.4, 154.7, 146.5, 136.9, 134.4, 133.3, 131.9, 131.7 131.6, 130.8, 129.7, 129.6, 128.9, 128.8, 127.0, 126.8, 124.9, 124.2, 123.9, 122.4, 121.2, 121.0, 120.6, 105.3, 31.2 ppm.

**4c**

| **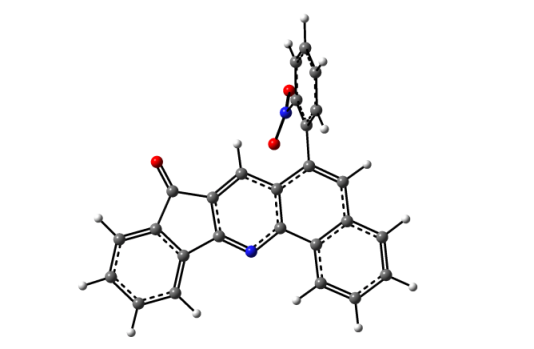** | **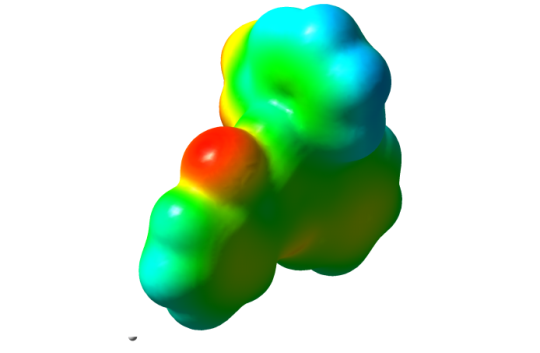** |
| --- | --- |
| **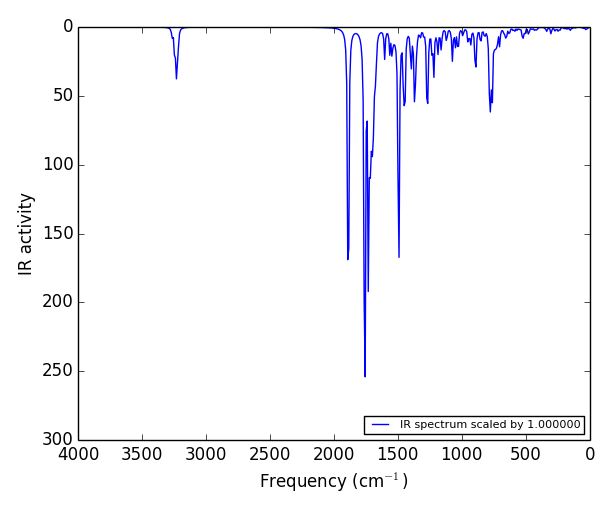** | |
| **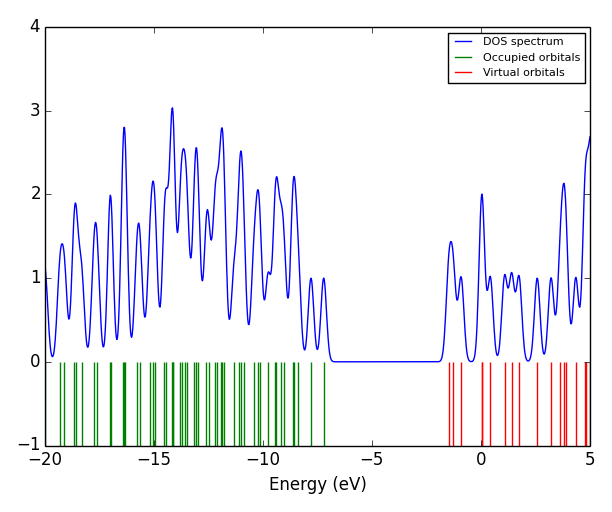** | |

**IR (KBr):** 3070 (C-H stretch, aromatic), 1713 (C=O stretch), 1607(C=N stretch), 1572, 1460, 1433 (C-C stretch, aromatic), 1520, 1350 (NO2 stretch), 1220 (C-CO-C bend), 856, 829, 792, 754, 735 (aromatic C-H out of plane bending) cm^-1^.

**^1^H NMR (400 MHz, DMSO-d_6_):** δ; 9.45 (d, *J* = 7.2 Hz, 1H, He), 8.45 (d, *J* = 8.0 Hz, 1H, Ha or Hd), 8.25 (d, *J* = 7.6 Hz, 1H, Hd or Ha), 8.10 (d, *J* = 6.8 Hz, 1H, Hh), 8.02-7.87 (m, 6H, Hc, Hj, Hk, Hm, Hl, Hn), 7.71-764 (m, 3H, Hb, Hf, Hg), 7.35 (d, *J* = 8.8 Hz, 1H, Hi) ppm.

**^13^C NMR (100 MHz, DMSO-d_6_):** δ; 191.1 (C=O), 162.5, 161.4, 154.1, 148.9, 148.3, 143.5, 136.7, 136.6, 134.9, 134.7, 132.6, 132.5, 131.2, 131.1, 130.3, 129.1, 128.7, 128.4, 125.6, 125.3, 124.6, 124.4, 123.5, 123.0, 122.0 ppm.

**4d**

| **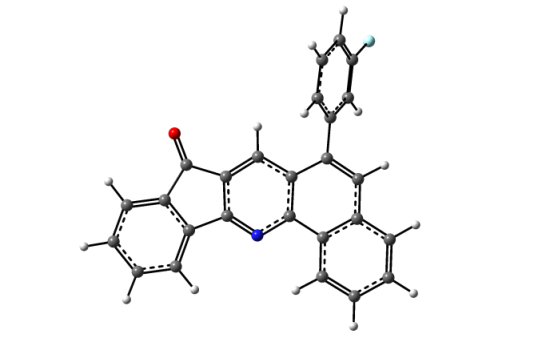** | **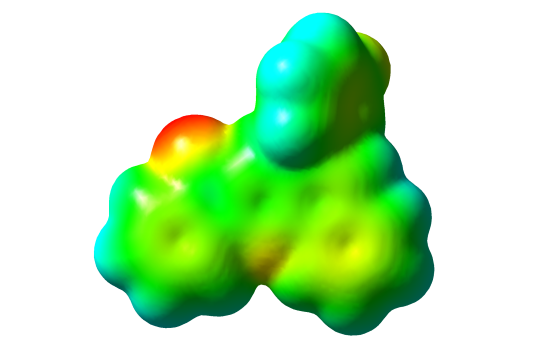** |
| --- | --- |
| **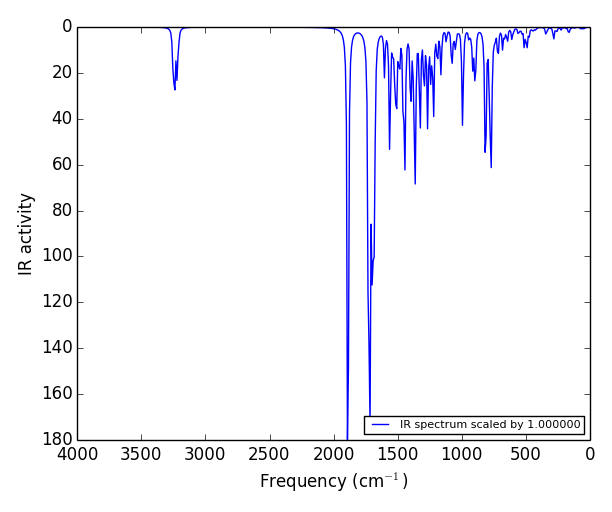** | |
| **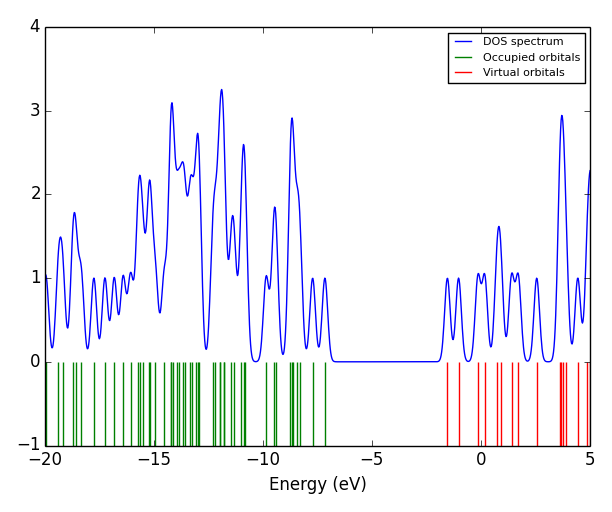** | |

**IR (KBr):** 3058 (C-H stretch, aromatic), 1712 (C=O stretch), 1606, 1575, 1517, 1477 (C-C stretch, aromatic), 1261 (C-F stretch), 1207 (C-CO-C bend), 871, 835, 794, 754, 694 (aromatic C-H out of plane bending) cm^-1^.

**^1^H NMR (400 MHz, DMSO-d_6_):** δ; 9.4 (dd, *J* = 7.6, 2.4 Hz, 1H, He), 8.19 (d, *J* = 7.2, 1H, Ha or Hd), 8.05 (dd, *J* = 6.4, 2.8 Hz, 1H, Hd or Ha), 7.91 (d, *J* = 4.4 Hz, 1H, Hh), 7.87-7.81 (m, 3H, Hf, Hg, Hj), 7.69-7.59 (m, 3H, Hb, Hc, Hk), 7.46 (m, 3H, Hl, Hm, Hn), 7.34 (d, *J* = 7.6 Hz, 1H, Hi) ppm.

**^13^C NMR (100 MHz, DMSO-d_6_):** δ; 190.0 (C=O), 162.0, 145.0, 143.1, 136.9, 136.8, 136.4, 134.6, 132.5, 131.2, 130.8, 130.7, 130.2, 128.7, 128.6, 128.2, 126.2, 125.6, 124.9, 124.2, 123.6, 123.9, 121.9, 117.2 (2JC-F = 21.0 Hz), 116.1 (2JC-F = 20 Hz) ppm.

**4e**

| **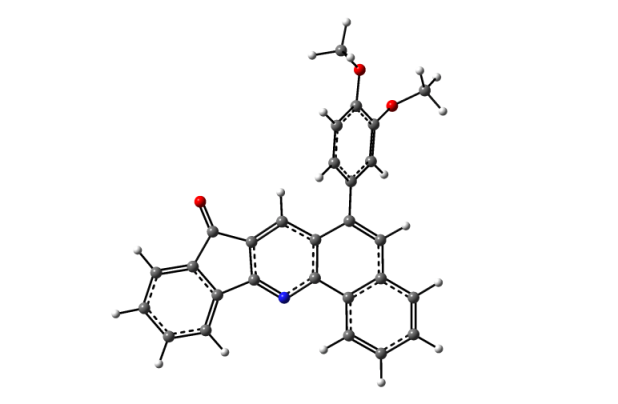** | **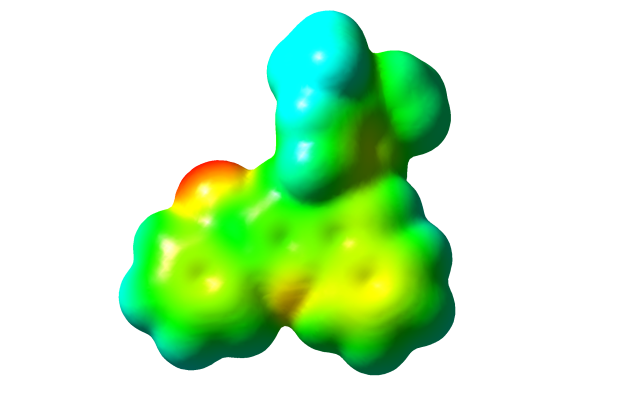** |
| --- | --- |
| **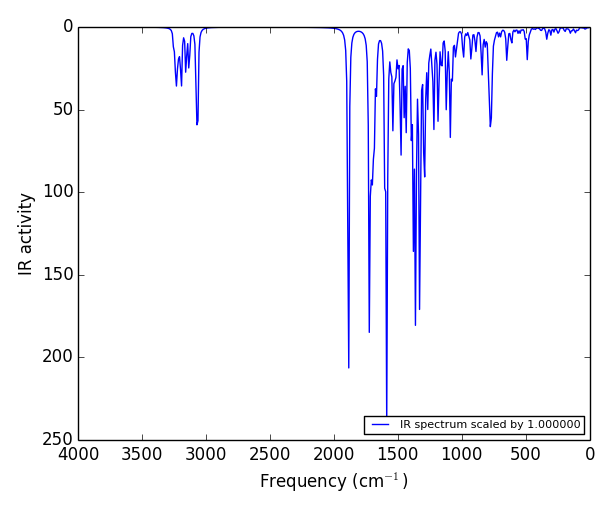** | |
| **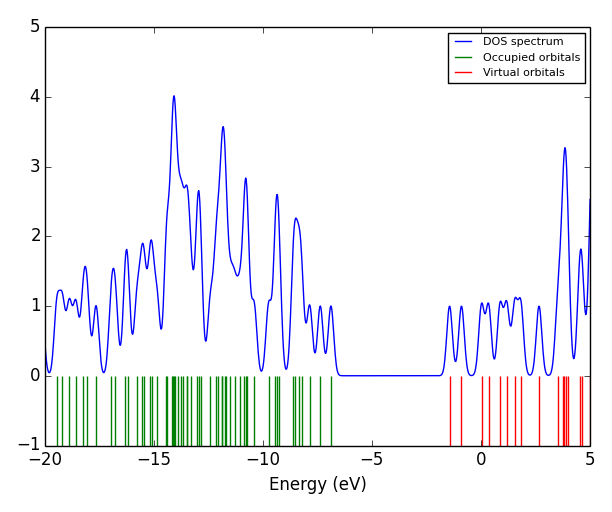** | |

**IR (KBr):** 3058 (C-H stretch, aromatic), 1712 (C=O stretch), 1604, 1570, 1510, 1460, (C-C stretch, aromatic), 1254, 1225, 1023 (C-O-C asymmetric and symmetric stretch, Ar-O-CH3), 872, 810, 751 (aromatic C-H out of plane bending) cm^-1^.

**^1^H NMR (400 MHz, DMSO-d_6_):** δ; 9.43 (d, *J* = 8.0 Hz, 1H, He), 8.23 (d, *J* = 7.2 1H, Ha or Hd), 8.07 (dd, *J* = 8.6, 3,6 Hz, 1H, Hd or Ha), 7.95 (d, *J* = 9.2 Hz, 1H, Hh), 7.89-784 (m, 3H, Hc, Hf, Hj), 7.72-7.63 (m, 3H, Hg, Hb, Hi), 7.18 (m, 2H, Hk, Hl), 7.06 (dd, J = 8.6, 3.6 Hz, 1H, Hm), 3.92 (s, 3H, OMe), 3.79 (s, 3H, OMe) ppm.

**^13^C NMR (100 MHz, DMSO-d_6_):** δ; 191.6 (C=O), 151.5, 149.8, 148.9, 147.7, 143.2, 140.0, 137.06, 137.0, 136.2, 134.6, 131.3, 130.1, 128.4, 128.3, 128.0, 125.6, 125.4, 124.5, 124.1, 122.9, 121.8, 115.2, 115.1, 114.3, 111.7, 56.2 (OMe), 56.0 (OMe) ppm.

**4f**

| **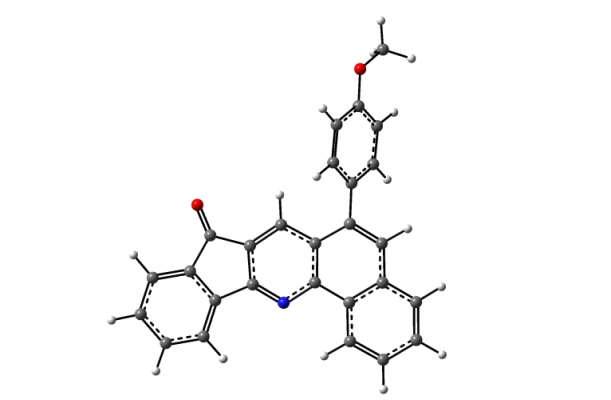** | **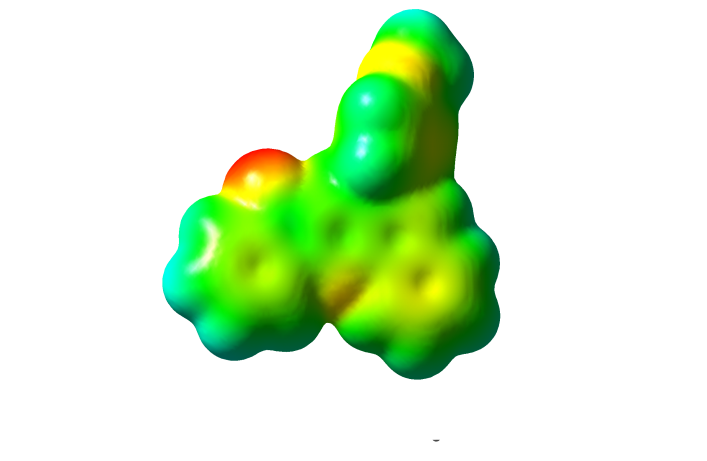** |
| --- | --- |
| **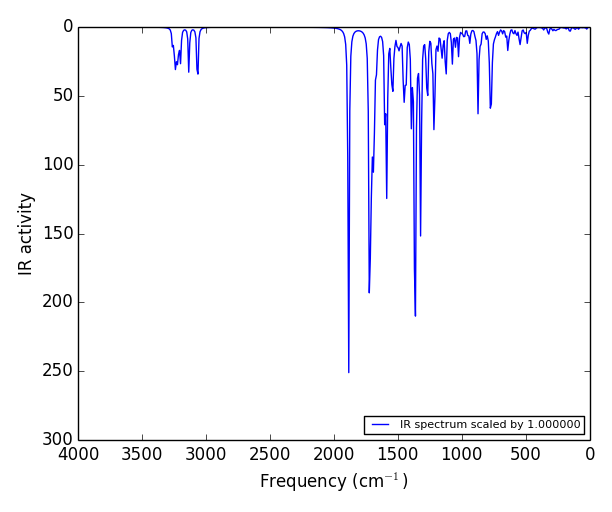** | |
| **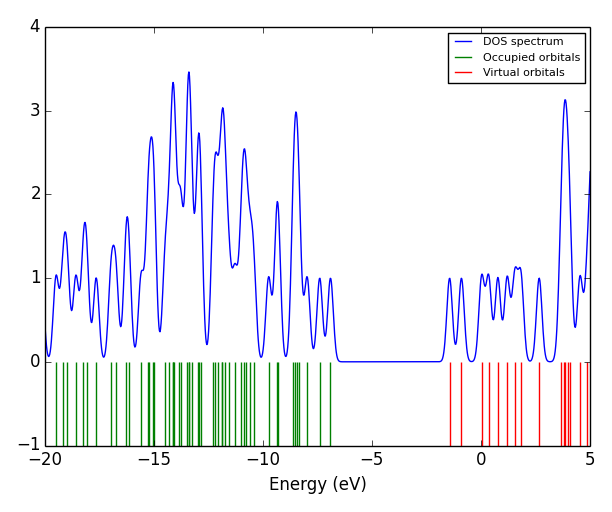** | |

**IR (KBr):** 3051 (C-H stretch, aromatic), 1713 (C=O stretch), 1608, 1569, 1508, 1492, 1458, (C-C stretch, aromatic), 1247, 1026 (C-O-C asymmetric and symmetric stretch, Ar-O-CH3), 839, 812, 758 (aromatic C-H out of plane bending) cm^-1^.

**4g**

| **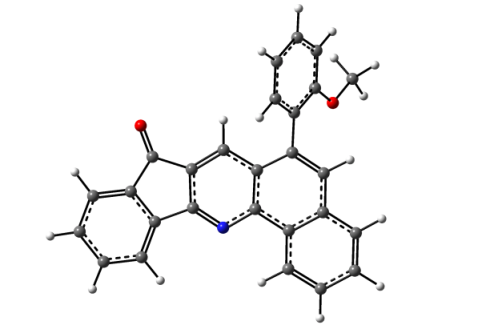** | **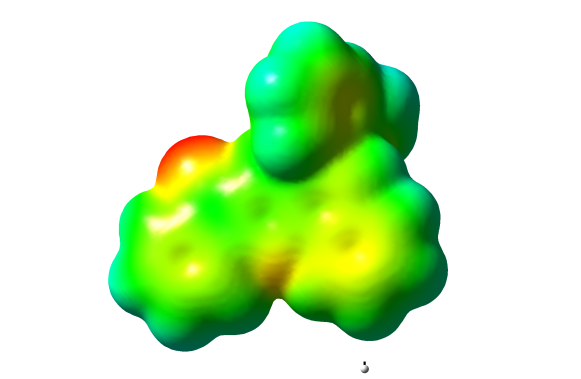** |
| --- | --- |
| **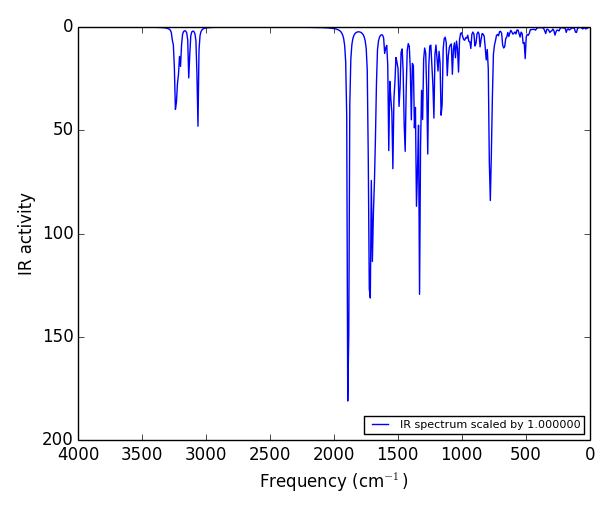** | |
| **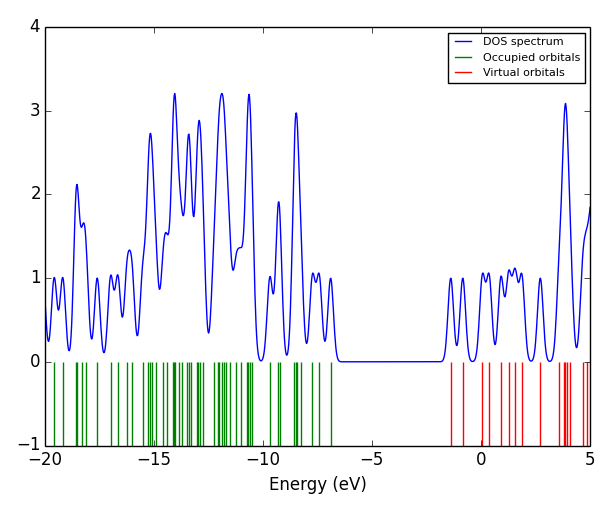** | |

**IR (KBr):** 3053, 3022 (C-H stretch, aromatic), 1706 (C=O stretch), 1608, 1575, 1485, 1458 (C-C stretch, aromatic), 1234, 1024 (C-O-C asymmetric and symmetric stretch, Ar-O-CH3), 875, 839, 810, 754 (aromatic C-H out of plane bending) cm^-1^.

**4h**

| **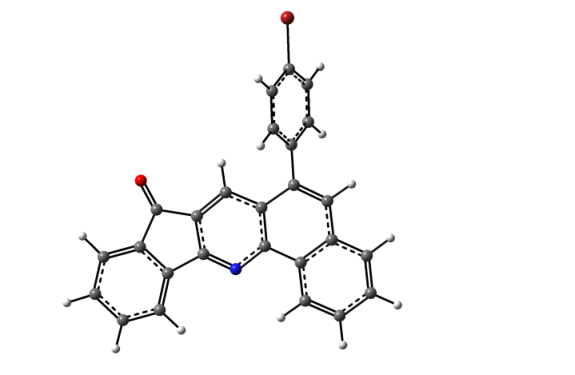** | **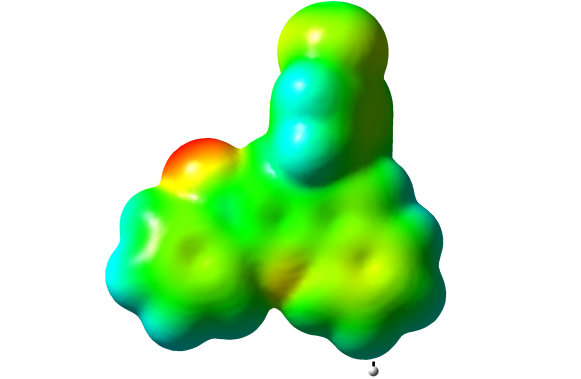** |
| --- | --- |
| **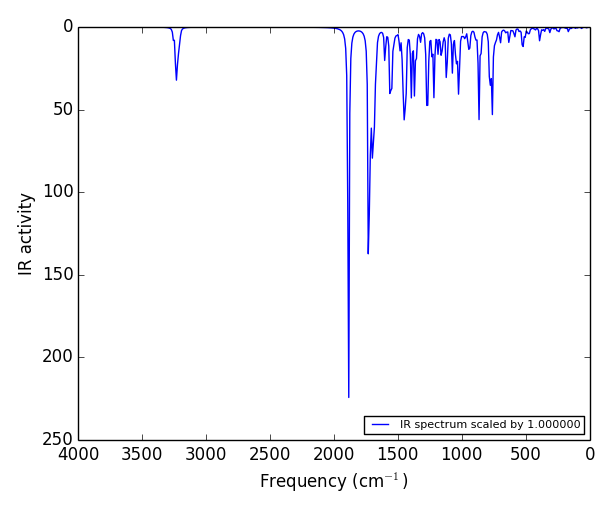** | |
| **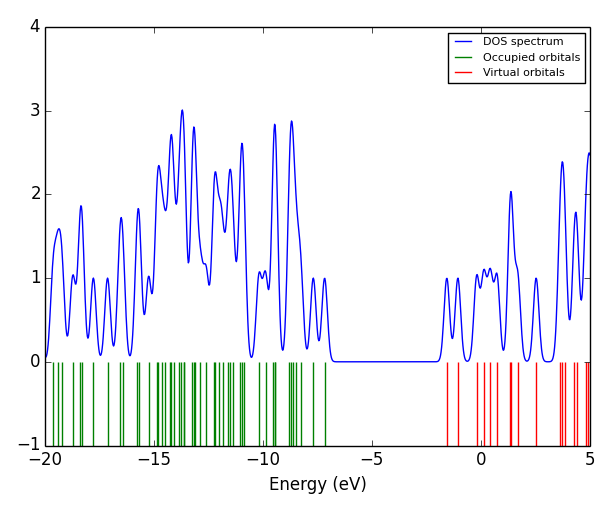** | |

**IR (KBr):** 3060 (C-H stretch, aromatic), 1709 (C=O stretch), 1603, 1574, 1519, 1479 (C-C stretch, aromatic), 1000 (C-Br stretch), 877, 838, 811, 753 (aromatic C-H out of plane bending) cm^-1^.

**4i**

|  |  |
| --- | --- |
|  | |
|  | |

**IR (KBr):** 3057 (C-H stretch, aromatic), 1715 (C=O stretch), 1607, 1575, 1520, 1468, (C-C stretch, aromatic), 1100, 1062 (C-Cl stretch), 874, 847, 800, 752 (aromatic C-H out of plane bending) cm^-1^.

**4j**

|  |  |
| --- | --- |
|  | |
|  | |

**IR (KBr):** 3059 (C-H stretch, aromatic), 1709 (C=O stretch), 1603, 1576, 1518, 1484, (C-C stretch, aromatic), 1087 (C-Cl stretch), 879, 840, 812, 749 (aromatic C-H out of plane bending) cm^-1^.

**4k**

|  |  |
| --- | --- |
|  | |
|  | |

**IR (KBr):** 3057 (C-H stretch, aromatic), 1715 (C=O stretch), 1604, 1574, 1516, 1470, (C-C stretch, aromatic), 1077 (C-Cl stretch), 860, 802, 752, 720 (aromatic C-H out of plane bending) cm^-1^.

**4l**

|  |  |
| --- | --- |
|  | |
|  | |

**IR (KBr):** 3055 (C-H stretch, aromatic), 1711 (C=O stretch), 1610, 1574, 1520, 1470, (C-C stretch, aromatic), 1036 (C-Cl stretch), 835, 810, 752, 704 (aromatic C-H out of plane bending) cm^-1^.

**References**

1. Mamaghani, M. & Larghani, T. H. Ultrasound promoted one-pot three-component synthesis of novel 7-aryl-8H-benzo[h]indeno[1,2-b]quinolin-8-ones under solvent-free conditions. *J. Chem. Res.* **36**, 235–237 (2012).

2. Mansoor, S. S.; Ghashang, M. Facile one-pot synthesis of a novel series of 7aryl-8Hbenzo[h]indeno[1,2-b]quinoline-8-one derivatives catalyzed by tribromomelamine. *Res. Chem. Intermed.* **41**, 6907-6926. (2014).

3. R. Sandaroos, M. Vadi, S. D. Efficient example of cross-linked polymeric catalysed synthesis of 7H-benzo[h]indeno[1,2-b]quinolin-8-one and 8H-naphtho[2,3-h]indeno[1,2-b]quinolin-9-one. *J. Chem. Sci.* **125**, 1497–1501 (2013).

4. Tasi, G., Palinko, I., Nyerges, L., Fejes, P. & Foerster, H. Calculation of electrostatic potential maps and atomic charges for large molecules. *J. Chem. Inf. Comput. Sci.* **33**, 296–299 (1993).
